# High CD206 levels in Hodgkin lymphoma‐educated macrophages are linked to matrix‐remodeling and lymphoma dissemination

**DOI:** 10.1002/1878-0261.12616

**Published:** 2020-01-28

**Authors:** Annekatrin Arlt, Frederike von Bonin, Thorsten Rehberg, Paula Perez‐Rubio, Julia C. Engelmann, Katharina Limm, Sarah Reinke, Christian Dullin, Xueni Sun, Rieke Specht, Markus Maulhardt, Franziska Linke, Gertrude Bunt, Wolfram Klapper, Martina Vockerodt, Jörg Wilting, Tobias Pukrop, Katja Dettmer, Wolfram Gronwald, Peter J. Oefner, Rainer Spang, Dieter Kube

**Affiliations:** ^1^ Clinic of Hematology and Medical Oncology University Medical Centre Göttingen Germany; ^2^ Network BMBF eMed MMML‐Demonstrators Regensburg Germany; ^3^ Statistical Bioinformatics Institute of Functional Genomics University of Regensburg Germany; ^4^ Institute of Functional Genomics University of Regensburg Germany; ^5^ Department of Pathology, Hematopathology Section UKSH Campus Kiel Germany; ^6^ Institute of Diagnostic and Interventional Radiology University Medical Centre Göttingen Germany; ^7^ Clinical Optical Microscopy Institute of Neuropathology University Medical Centre Göttingen Germany; ^8^ Institute of Anatomy and Cell Biology University Medical Centre Göttingen Germany; ^9^ Department of Internal Medicine III, Hematology and Medical Oncology University Hospital Regensburg Germany; ^10^ Present address: NIOZ Royal Netherlands Institute for Sea Research Utrecht University Utrecht The Netherlands; ^11^ Present address: School of Medicine Queen’s Medical Centre Campus University of Nottingham UK

**Keywords:** CD206, lymphoma, macrophages, tumor microenvironment

## Abstract

Macrophages (Mφ) are abundantly present in the tumor microenvironment and may predict outcome in solid tumors and defined lymphoma subtypes. Mφ heterogeneity, the mechanisms of their recruitment, and their differentiation into lymphoma‐promoting, alternatively activated M2‐like phenotypes are still not fully understood. Therefore, further functional studies are required to understand biological mechanisms associated with human tumor‐associated Mφ (TAM). Here, we show that the global mRNA expression and protein abundance of human Mφ differentiated in Hodgkin lymphoma (HL)‐conditioned medium (CM) differ from those of Mφ educated by conditioned media from diffuse large B‐cell lymphoma (DLBCL) cells or, classically, by macrophage colony‐stimulating factor (M‐CSF). Conditioned media from HL cells support TAM differentiation through upregulation of surface antigens such as CD40, CD163, CD206, and PD‐L1. In particular, RNA and cell surface protein expression of *mannose receptor 1* (*MRC1*)/CD206 significantly exceed the levels induced by classical M‐CSF stimulation in M2‐like Mφ; this is regulated by interleukin 13 to a large extent. Functionally, high CD206 enhances mannose‐dependent endocytosis and uptake of type I collagen. Together with high matrix metalloprotease9 secretion, HL‐TAMs appear to be active modulators of the tumor matrix. Preclinical *in ovo* models show that co‐cultures of HL cells with monocytes or Mφ support dissemination of lymphoma cells via lymphatic vessels, while tumor size and vessel destruction are decreased in comparison with lymphoma‐only tumors. Immunohistology of human HL tissues reveals a fraction of cases feature large numbers of CD206‐positive cells, with high *MRC1* expression being characteristic of HL‐stage IV. In summary, the lymphoma‐TAM interaction contributes to matrix‐remodeling and lymphoma cell dissemination.

## Graphical abstract

The study highlights the ability of Hodgkin lymphoma cells to attract and to differentiate monocytes into M2‐like Mφ and the role of IL13 in regulating CD206 but also that CD206 contributes to the remodeling of the tumor microenvironment via take up glycoconjugates as well as type‐I collagen and immunosuppression by PD‐L1 upregulation.

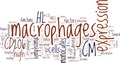

**Abbreviations**
APC: allophycocyanin
CAM: chick chorioallantoic membrane
CM: conditioned medium
DDA: data‐dependent acquisitions mode
DLBCL: diffuse large B‐cell lymphoma
DSMZ: Deutsche Sammlung für Mikroorganismen und Zelllinien
FACS: fluorescence‐activated cell sorting
FFPE: formalin‐fixed paraffin‐embedded
HL: Hodgkin lymphoma
HRP: horseradish peroxidase
M‐CSF: macrophage colony‐stimulating factor
MMP: matrix metalloprotease
MRC1: mannose receptor 1
Mφ: macrophages
PBMC: peripheral blood mononuclear cells
PE: phycoerythrin
PTA: phosphotungstic acid
RPMI: Roswell Park Memorial Institute
TAM: tumor‐associated Mφ
TMA: tissue microarray
TME: tumor microenvironment

## 1 Introduction

The tumor microenvironment (TME) plays an important role in numerous malignancies and may even provide a tumor supportive milieu. The TME contains a wide variety of cytokines and growth factors and supports crosstalk between neighboring cells. Among the stromal cells found in the TME, tumor‐attracted and re‐educated macrophages (Mφ), also designated as tumor‐associated Mφ (TAM), have received increasing interest (Mantovani and Sica, 2010; Twum *et al.*, 2017). They are involved in various aspects of tumor progression like aberrant cytokine secretion, immune check point modulation, tumor matrix remodeling, angiogenesis, and resistance to treatment (Mantovani *et al.*, 2017, 2002; Mantovani and Sica, 2010). TAMs have been implicated in the pathogenesis of various lymphoma subtypes such as Hodgkin lymphoma (HL), chronic lymphatic leukemias, follicular lymphoma, and aggressive non‐ HLs such as Burkitt lymphoma and DLBCL (Kridel *et al.*, 2015b, 2015a; Lenz *et al.*, 2008; Pham *et al.*, 2018; Steidl *et al.*, 2011; Steidl *et al.*, 2010; Verdiè *et al.*, 2018).

Hodgkin lymphoma is a paradigm of a TME‐dominated tumor that is characterized by a very small portion (< 1%) of malignant cells surrounded by abundant supportive cells, among which T cells often dominate (Aldinucci *et al.*, 2010; Küppers, 2009; Scott and Steidl, 2014; Steidl *et al.*, 2011). Additionally, Mφ are an integral part of the TME, and the number of CD68‐positive Mφ predicts the patient’s outcome (Scott and Steidl, 2014). Therefore, targeting the interaction between HL cells and Mφ presents a promising strategy in the treatment of HL (Locatelli *et al.*, 2018; Ruella *et al.*, 2017). However, our knowledge of the functions and interactions of Mφ in HL is still limited (Mantovani *et al.*, 2002; Martinez and Gordon, 2014; Steidl *et al.*, 2010). The present study sheds new light on the molecular interaction between HL cells and TAMs and shows that HL‐educated TAMs are capable of supporting tissue remodeling and lymphoma dissemination involving the mannose receptor 1 (*MRC1*/CD206).

## 2 Materials and methods

### 2.1 Cell lines, conditioned media, reagents, and functional assays

The HL cell lines L428, KM‐H2, L1236, L540, and HDLM‐2 were provided by V. Diehl (Cologne). The DLBCL cell lines HBL‐1 and OCI‐LY3 were obtained from D. Krappmann (Munich) and the Deutsche Sammlung für Mikroorganismen und Zelllinien (DSMZ; Braunschweig, Germany), respectively. All cell lines were maintained in Roswell Park Memorial Institute (RPMI) 1640 supplemented with 2 mg·mL^−1^ glutamine and 10% FCS. At regular intervals, we used a highly specific Ig PCR to test for cell identity. In addition, L428, L1236, KM‐H2, OCI‐Ly3, and HBL1 cells had been subjected recently to STR profiling by the DSMZ (Braunschweig, Germany).

For the production of lymphoma‐conditioned media (CM), cells were seeded at a density of 5 × 10^5^ cells per mL and incubated in complete RPMI 1640 medium for 2 days. Cell supernatants were centrifuged at 300 ***g*** for 10 min at 4 °C, sterile‐filtered, and stored at 4 °C for a maximum of 2 weeks.

#### 2.1.1 Monocyte isolation

Peripheral blood mononuclear cells (PBMCs) of healthy donors were isolated from fresh buffy coats by density‐gradient centrifugation over Biocoll separating solution (Biochrom, Berlin, Germany). CD14^+^ monocytes were obtained from PBMCs by magnetic cell separation using CD14 microbeads (Miltenyi Biotec, Bergisch Gladbach, Germany) according to the manufacturer’s instructions. Purity of CD14^+^ cells after magnetic cell separation was determined by staining with specific markers and quantification by flow cytometry using a FACSCanto II (BD Biosciences, Franklin Lakes, NJ, USA).

#### 2.1.2 Macrophage differentiation

Monocyte isolation and macrophage differentiation were performed as described previously (Menck *et al.*, 2014). Monocytes were differentiated either in the presence of RPMI 1640 containing 10% (v/v) FCS, Pen/Strep, and 2.5 ng·mL^−1^ recombinant M‐CSF (Immunotools, Friesoythe, Germany) or in lymphoma‐CM mixed in equal parts with complete RPMI1640. Briefly, M‐CSF or CM of the tested lymphoma cell lines was used to incubate primary human monocytes for 7 days in Teflon‐coated cell culture equipment. After 7 days, cells were counted, harvested, and transferred into cell culture dishes for additional functional analysis. Freshly isolated monocytes or Mφ were stained with cell‐specific antibodies or isotype controls. Expression was quantified by flow cytometry using a fluorescence‐activated cell sorting (FACS) Canto II and calculated by dividing the mean fluorescence intensity (MFI) by the MFI of the corresponding isotype control. For intracellular CD68 staining, cells were fixed and permeabilized using the Cytofix/Cytoperm Kit (BD BioSciences) following the manufacturer’s instructions.

For migration assays, a Boyden chamber with a 5‐µm porous membrane was used (Neuro Probe Inc., Gaithersburg, MD, USA). 5 × 10^4^ monocytes were seeded per well in 50 µL of RPMI 1640 medium and allowed to migrate for 2 h toward RPMI 1640 supplemented with 1% or 10% (v/v) FCS or lymphoma‐ CM. The cells that had migrated into the lower chamber were counted.

To analyze endocytosis, Mφ were allowed to adhere overnight on tissue culture dishes. Cells were washed twice with PBS and maintained in RPMI 1640 containing 10% (v/v) FCS and pen/strep. 10‐ and 70‐kDa FITC dextran (at 1 mg·mL^−1^ for 2 h; Sigma Aldrich, Munich, Germany), respectively, or Gelatin Oregon Green™ 488 Conjugate (at 5 µg·mL^−1^ for 30 min; Thermo Scientific, Waltham, MA, USA) were added to each plate and incubated at 37 °C or on ice. After incubation, cells were washed twice with PBS, harvested using trypsin/EDTA, and fixed in 2% formaldehyde solution in PBS. Uptake was analyzed by means of a FACS Canto II and calculated by dividing the MFI of the 37 °C sample by the corresponding MFI of the sample incubated on ice.

The activity of matrix metalloproteinase (MMP)‐9 was determined in cell culture supernatants by gelatin zymography. First, proteins were separated by SDS/PAGE. Gel mixes were used to prepare 8% acrylamide gels containing 1% gelatin and 5% acrylamide stacking gels. Fifteen microliters of cell culture supernatant has mixed with an equal volume of loading buffer (62.5 mm Tris/HCl, pH 6.8, 4% SDS, 25% glycerol, 0.01% bromophenol blue) and loaded onto the gel. Electrophoresis was performed under cooling using freezer packs. Afterward gels were incubated in wash buffer (50 mm Tris/HCl, pH 7.5, 5 mm CaCl_2_, 2.5% Triton X‐100) for 1 h. Gels were transferred to renaturation buffer (2.5% Triton X‐100) and incubated 1 h. Afterward gels were covered in development buffer (50 mm Tris base, 150 mm NaCl, 10 mm CaCl_2_) and incubated overnight at 37 °C with soft agitation. To visualize the gel degradation by MMP activity, the gels were then stained in staining buffer (0.5% Coomassie blue, 40% methanol, 10% acetic acid) for 1 h, followed by destaining in destaining buffer (40% methanol, 10% acetic acid) for 1.5 h. Gels were then fixed for 30 min in fixation buffer (5% glycerol, 30% methanol), placed between two cellophane membranes and dried overnight. Fixed and dried gels were scanned for image processing.

Recombinant M‐CSF (ImmunoTools) and IL‐13 (PeproTech, Hamburg, Germany) were used at 2.5 and 10 ng·mL^−1^, respectively. Inhibitors: the pan‐JAK inhibitor pyridone‐6 was obtained from Merck (Darmstadt, Germany), while ruxolitinib, BKM120 (NVP‐BKM120), and ibrutinib ((PCI‐32765) were purchased from Absource Diagnostics GmbH (Munich, Germany).

#### 2.1.3 Fluorescence‐labeled antibodies

FITC mouse anti‐CD1a (HI149), FITC mouse anti‐CD11b (LT11), FITC mouse anti CD11c (BU15), FITC mouse anti‐CD31 (MEM‐05), FITC mouse anti‐CD33 (HIM3‐4), FITC mouse anti‐CD40 (HI40a), FITC mouse anti‐CD44 (MEM‐85), FITC mouse anti‐CD54 (1H4), FITC mouse anti‐CD80 (MEM‐233), FITC mouse anti‐CD86 (BU63; ImmunoTools), FITC mouse anti‐CD14 (M5E2), FITC mouse anti‐HLA‐DR (G46‐6), phycoerythrin (PE) mouse anti‐CD68 (Y1/82A; BD Bioscience), allophycocyanin (APC) mouse anti‐CD163 (GHI/61), APC mouse anti‐CD206 (15‐2), APC mouse anti‐PDL1 (29E.2A3; BioLegend, San Diego, CA, USA) and isotype controls FITC mouse IgG1 (PPV‐06), FITC mouse IgG2b (PLRV219; ImmunoTools), FITC mouse IgG2a (G155‐178), PE mouse IgG2b (27‐35; BD BioScience), APC mouse IgG2b (MPC‐11; BioLegend) were used for flow cytometry.

Primary mouse anti‐CD30 (Ber‐H2) and mouse anti‐CD68 (KP1), anti‐Prox1, anti‐CD206 (D‐1; Santa Cruz Biotechnology Inc., Dallas, TX, USA), and secondary goat anti‐mouse horseradish peroxidase (HRP) polyclonal; Agilent, Santa Clara, CA, USA) were used for peroxidase and immunofluorescence staining.

ELISA kits for the detection of secreted M‐CSF and IL‐13 in cell culture supernatants were purchased from R&D Systems (Minneapolis, MN, USA). The sensitivity was 11.2 and 13.2 pg·mL^−1^, respectively.

#### 2.1.4 Immunohistochemistry on FFPE sections and immunofluorescence on cryotissue sections

Conventional immunohistochemical staining of CD30, CD68, and CD206 was carried out according to standard procedures including endogen peroxidase blocking (10 min in methanol/H_2_O_2_) and antigen retrieval (3 min at 100 °C in 10 mm citrate buffer, pH 6.0) prior to incubation with the antibodies. Detection was performed using the ZytoChemPlus (HRP) Polymer Bulk Kit (Zytomed Systems GmbH, Berlin, Germany). The counterstaining was performed with Hemalaun after Meyer (1 : 4; Merck) for 3–5 min and rinsing with water for 10 min. After dehydration, the sections were covered with the mounting medium Pertex (Histolab Products AB; Göteborg, Sweden). Immunofluorescence detection of Prox1 (1 : 500, Reliatech, Wolfenbüttel, Germany), CD30, and CD68 (Dako/Agilent, Hamburg Germany) was performed on 12‐µm cryosections. Appropriate Alexa Fluor®‐conjugated secondary antibodies were used (1 : 200; Life Technologies, Eugene, OR, USA). Immunofluorescence staining of CD163 and CD206 in tonsil and HL tissue sections was performed using anti‐CD163 antibody (1 : 20, AM10011PU‐S rabbit, ACRIS/ OriGene Technologies GmbH, Herford, Germany) or the above‐mentioned anti‐CD206 antibody (1 : 100) in Antibody Diluent (Medac GmbH, Wedel, Germany), and subsequent staining with corresponding secondary antibody (Donkey anti‐mouse Alexa 555 and Donkey anti‐rabbit Alexa 488 [1 : 100, Invitrogen/Thermo Fisher Scientific GmbH, Dreieich, Germany)] and 4′,6‐diamidino‐2‐phenylindole (1 : 5000, Invitrogen/Thermo Fisher Scientific GmbH). For each staining step, 100 µL of the respective dilution pro object slide was used. The anti‐CD206 staining of TMAs was performed with the above‐mentioned anti‐CD206 antibody and Histofine anti‐Mouse Histofine anti‐mouse and anti‐rabbit (Medac GmbH) and stained with 3,3'‐diaminobenzidine (Dako/Agilent Germany). For tissue microarray (TMA) staining, 200 µL of each dilution was used.

Slides were scanned by a Hamamatsu Nanozoomer 2.0 RS slide scanner (Hamamatsu Photonics, Ammersee, Germany) with 40× magnification for the TMAs and a 20× magnification for the fluorescent‐labeled slides of HL and tonsils. Raw image data were saved in.ndpi format and handled by the software kit ndp.view 2 (Hamamatsu Photonics) to save details of the whole image in.jpeg format. Figures were made with Adobe Photoshop CS4 Extended (Adobe Systems Incorporated, San Jose, CA, USA). Chick chorioallantoic membrane (CAM) tissue slides were scanned with a 20× objective (UPlanSApo, NA 0.75) using the virtual slide microscope VS120‐L100 (Olympus, Hamburg, Germany) equipped with a VC‐50 camera.

The experiments and analysis were undertaken with the understanding and written consent of each subject. The study methodologies conformed to the standards set by the Declaration of Helsinki. The study methodologies were approved by the local ethics committee [Medical Faculty of the Georg‐August‐University Göttingen (No. 16/5/18An); Medical Faculty of the Christian‐Albrechts‐University Kiel, Germany (No. 447/10)].

### 2.2 Chick chorioallantoic membrane (CAM) assay

Chicken eggs (Valo BioMedia GmbH, Osterholz‐Scharmbeck, Germany) were bred and regularly turned for 3 days at 37.8 °C and 80% humidity as described recently (Klingenberg *et al.*, 2014a; Klingenberg *et al.*, 2014b).On day 3, a window was sawed into the egg and sealed with sellotape. On day 10, 2 × 10^6^ L428 cells with or without 1 × 10^6^ CD14^+^ PBMCs or corresponding 1 × 10^6^ Mφ embedded in 20 µL Matrigel (BD Biosciences) were inoculated onto the CAM. After 4 days of incubation, tumors were cut out, fixed, and embedded in paraffin or Tissue Tek (PolySciences Inc., Warrington, PA, USA) and cut into 3‐ or 11‐µm‐thick sections, respectively.

In order to access tumor volume and blood content, specimens were stained with phosphotungstic acid (PTA) according to the protocol described recently (Dullin *et al.*, 2017). Then, they were embedded in 1% agarose gel in 2‐mL plastic tubes and scanned using the *in vivo* microcomputed tomography (micro‐CT) QuantumFX (Perkin Elmer Health Sciences, Hopkinton, MA, USA) and the following acquisition parameters: 90‐kV tube voltage, 200‐µA tube current, FOV 20 × 20 mm^2^, 2‐min total acquisition time resulting in 3D datasets with a voxel size of 40 × 40 × 40 µm^3^. The software scry v6.0 (Kuchel & Sautter GbR, Rötenbach, Germany) was used for 3D rendering and volume measurement. For this purpose, the CAM around the tumor was manually removed using a virtual scalpel and the tumor mass was segmented based on a brightness threshold.

### 2.3 Transcriptomics

The samples were analyzed by RNA‐Seq. Read quality was assessed with fastQC (Andrews (2010): FastQC: a quality control tool for high‐throughput sequence data. Available online at: https://www.bioinformatics.babraham.ac.uk/projects/fastqc/), FastqPuri and QoRTs (Hartley and Mullikin, 2015; Perez‐Rubio *et al.*, 2018). First, we removed reads perfectly matching the human ribosomal repeating unit (GenBank accession U13369.1) with FastqPuri. Sequences were then filtered and trimmed based on quality scores (bases with quality scores below 28 were removed from the ends and the remaining read accepted if it had fewer than 5% base qualities below 28). Then, undetermined bases (Ns) were removed from the reads and the largest N‐free sequence was kept as long as it was at least 25 nt long. Transcript counts were generated with Kallisto using the Ensembl *homo sapiens* genome (release 87). The mean pseudo‐alignment rate was 87.42%. In order to mitigate the donor effect, the combat function of the R‐package sva was applied (Leek J.T. (2018): Available online at: http://bioconductor.org/packages/release/bioc/html/sva.html). DESeq2 was used for differential gene expression (GE) analysis and *P*‐values were adjusted for multiple testing using the false discovery rate (Hartley and Mullikin, 2015). KEGG pathway analysis was performed using the R‐package gage (Luo *et al.*, 2009). Variance stabilizing transformation implemented in the R‐package DESeq2 was applied to normalize the data for t‐SNE plots. Subsequently, t‐SNE dimension reduction was performed using the R‐package Rtsne (Maaten and Hinton, 2008; Krijthe (2015): Available online at: https://cran.r-project.org/web/packages/Rtsne/citation.html). GE data are available at NCBI project number PRJNA592308.

### 2.4 Proteomics

The samples (25 µg per replicate) were subjected to tryptic digestion using the GASP protocol (Fischer and Kessler, 2015). Five micrograms of the resulting peptide mixtures were spiked with 100 fmol of the retention time standard RePLiCal (Polyquant GmbH, Bad Abbach, Germany) and analyzed using an Eksigent ekspert™ nanoLC 400 system coupled to a TripleTOF 5600^+^ mass spectrometer (SCIEX, Framingham, MA, USA).

Five microliters of sample was loaded onto a YMC‐Triart C18 trap column (3 μm particle size, 0.5 cm length; YMC America, Inc., Allentown, PA, USA) at a flow‐rate of 10 µL·min^−1^ for 5 min (isocratic conditions A: 0.1% formic acid, 0.1% ACN). Peptides were then separated on a reverse‐phase column (YMC‐Triart C18, 1.9 µm particle size, 120 Å, flow‐rate of 5 µL·min^−1^, 40 °C) using a 94‐min binary acetonitrile gradient (3–40% B in 87 min, 40–45% B in 7 min).

The sequential window acquisition of all theoretical fragment‐ion spectra (SWATH) runs was accomplished using a 50 ms full‐MS scan from 400–1000 *m/z* and 60 subsequent SWATH windows of variable size for 35 ms each (mass range, 230–1500 *m/z*).

The respective libraries were generated from pooled samples measured in data‐dependent acquisitions mode (DDA) using the TOP20 method with a full‐MS scan for 250 ms and MS/MS scans for 50 ms each. MS/MS spectra from the DDA runs were searched against the respective UniProt database (swissprot‐human09‐2017) using ProteinPilot 5.0 and imported in PeakView 2.1 using the SWATH MicroApp 2.0 allowing six peptides per protein and five transitions per peptide. Raw values were normalized to total intensity.

The MS proteomics data and search output file have been deposited to the ProteomeXchange Consortium (Perez‐Riverol *et al.*, 2019) via the PRIDE partner repository with the dataset identifier PXD01612.

### 2.5 Combining transcriptome and proteome data

Filtering for matching proteins and genes in the combined transcriptome and proteome data yielded 1938 gene–protein pairs. Further filtering for genes and proteins that both differed (*P*_adj_ < 0.01) in expression between DLBCL and HL stimulated Mφ brought down the number to 13 gene–protein pairs (Table 1, top). Analogously, comparing HL and M‐CSF‐stimulated Mφ revealed 10 gene–protein pairs (Table 1, bottom). In both comparisons, CD206 was the most differential protein/gene with a log_2_ fold change (LFC) of ~ 3.

**Table 1:** List of genes that were significantly differentially regulated between L428‐educated Mφ and DLBCL‐ and M‐CSF‐educated Mφ, respectively, at both the gene and the protein level.

	LFC GE	adj.*P*‐value	LFC protein	adj.*P*‐value	Description
L428 vs. DLBCL					
ALOX5AP	3.7942	1.17E‐49	2.402749983	3.77E‐05	Arachidonate 5‐lipoxygenase‐activating protein
MRC1	3.0765	1.84E‐13	2.88006277	1.55E‐08	Macrophage MRC1
UPP1	1.7246	2.88E‐11	0.835701997	0.000141198	Uridine phosphorylase 1
EPHX1	0.97398	2.95E‐08	0.447050392	0.007422156	Epoxide hydrolase 1
HVCN1	0.94723	1.97E‐10	0.808504269	0.009561778	Voltage‐gated hydrogen channel 1
TTC39B	0.89962	1.56E‐06	0.758481005	0.003038771	Tetratricopeptide repeat protein 39B
IL1RN	0.7755	0.0027549	1.145794579	0.008503356	Interleukin‐1 receptor antagonist protein
TKT	0.69094	4.81E‐15	0.585160474	0.007700075	Transketolase
PTGR1	−0.35052	0.0074362	0.562922246	0.009561778	Prostaglandin reductase 1
TCIRG1	−0.50824	0.0004946	−0.524372957	0.009953695	V‐type proton ATPase
MAN2B1	−0.53211	9.46E‐06	−0.547919395	0.006243247	Lysosomal alpha‐mannosidase
CPT1A	−0.75596	4.83E‐08	−0.662185065	0.008503356	Carnitine O‐palmitoyltransferase 1
SRM	−0.93767	9.53E‐11	0.437485354	0.009953695	Spermidine synthase
L428 vs. M‐CSF					
MRC1	3.2116	1.14E‐09	3.011783544	4.53E‐10	Macrophage MRC1
ALOX5AP	2.0344	8.12E‐10	1.597124746	0.004082951	Arachidonate 5‐lipoxygenase‐activating protein
UPP1	1.6381	9.00E‐07	0.928778604	0.000540053	Uridine phosphorylase 1
ALDH2	1.6264	3.57E‐18	0.876242857	0.004334703	Aldehyde dehydrogenase 2 family member
STOM	1.3435	0.0009476	1.046572022	0.004082951	Stomatin
TTC39B	1.0793	6.08E‐06	0.908818503	0.001644753	Tetratricopeptide repeat domain 39B
EML4	1.0054	2.67E‐13	1.229500721	0.004082951	Echinoderm microtubule‐associated protein‐like 4
IL1RN	1.0028	0.0028429	1.427849353	0.001274962	Interleukin‐1 receptor antagonist protein
GCA	0.57588	0.0007919	1.034796929	0.001644753	Grancalcin
FUCA1	−0.76156	0.0073074	−1.154748957	0.001644753	Alpha‐l‐fucosidase 1

In addition, differential genes and proteins are listed in Tables [Link], [Link], [Link], [Link], [Link]: geneExpression_deseq_hl_vs_mcsf.csv, geneExpression_deseq_hl_vs_dlbcl.csv, proteomics_limma_hl_vs_mcsf.csv, proteomics_limma_hl_vs_dlbcl.csv, proteomics_limma_dlbcl_vs_mcsf.csv.

### 2.6 Statistical and regression analyses

Results are shown as mean or as mean ± SD of the indicated number of samples. The statistical significance of the values was determined using the Student's *t*‐test. If applicable, group results were compared by ANOVA (one‐way or two‐way analysis of variance) with subsequent Bonferroni’s *post hoc* test to correct for multiple comparisons as indicated. Normal distribution and homogeneity of variance were tested using the Kolmogorov–Smirnov test and the *F*‐test, respectively. The Kruskal–Wallis test with Dunn’s *post hoc* test was performed for nonparametric testing. Significance levels are indicated as *P < 0.05, **P < 0.01 and ***P < 0.001. All statistical analyses and plots were done using graphpad prism 6.04 (GraphPad Software Inc., La Jolla, USA).

## 3 Results

### 3.1 Hodgkin lymphoma cell supernatants attract monocytes and initiate their differentiation into Mφ characterized by high mannose receptor 1 expression

To gain insight into the capacity of lymphoma cells to attract and differentiate monocytes into Mφ, the migration of monocytes toward conditioned media (CM) from five HL and two DLBCL cell lines was first investigated by the Boyden chamber assay and compared to unconditioned medium containing 1% and 10% FCS, respectively. Only CM from HL cell lines, with the exception of HDLM‐2, exhibited significant chemo‐attractive activity (Fig. S1A). However, as exemplified by KMH2‐CM, strong chemo‐attractive activity does not necessarily translate into strong Mφ differentiation capacity (Fig. S1B). This suggests, in contrast to previous findings (Locatelli *et al.*, 2018; Ruella *et al.*, 2017), that attraction and differentiation are distinct processes regulated by different cytokines or chemokines.

**Figure 1:**
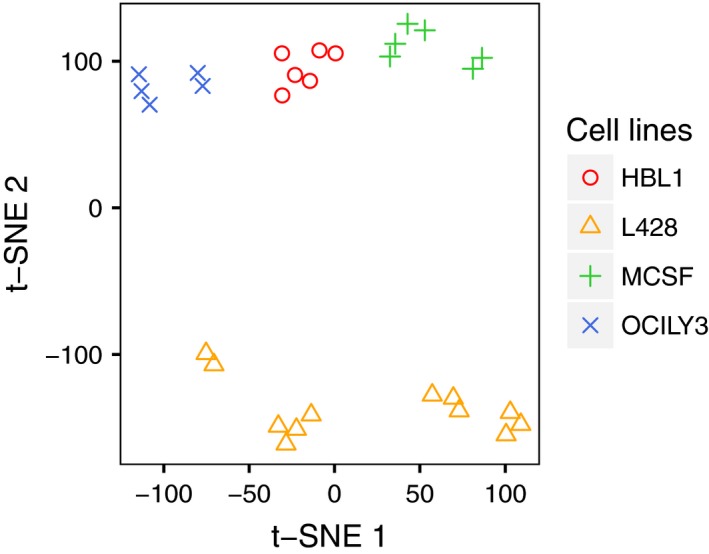
Factors secreted by HL cells induce distinct changes in global gene expression by Mφ. Shown is a t‐SNE plot to visualize differences in global gene expression between L428 (HL), OCI‐Ly3 (DLBCL), HBL‐1 (DLBCL), and M‐CSF‐induced Mφ.

Next, monocytes from three different donors were differentiated with L428‐CM, OCI‐Ly3‐CM, HBL1‐CM, and M‐CSF, respectively. GE of Mφ was analyzed by RNA‐Seq and compared between Mφ treated with L428‐CM, DLBCL‐CM, and M‐CSF, respectively (Fig. 1; Table S1 and S2). L428‐educated Mφ are distinct from DLBCL‐CM and M‐CSF‐differentiated Mφ (Fig. 1). They showed high expression levels of such typical macrophage markers as CD68 and *CD163*. Their expression levels matched those of *CCR1*, *CSF1*, *CSFR1*, *CTSH*, *CTSL*, *CXCL16*, *or CD53*, *CD164*, *CD276*, *ITGAX*, *TGM2*, *TGFB1*, and *TGFBI*, just to name a few. Among moderately expressed genes were, among others, *CD209* (DC‐SIGN), *CXCR4*, *CXCR5*, *IFNAR2*, *IFGR*, *IGF1R*, *IL16*, *IL6R*, *IL13RA*, *STAB1*, *S1PR2*, *SPHK1,* and *STAT1*, whereas *ADGRE2*, *CCL28*, *CXCL10*, *CXCL11*, *CX3CL1*, *CXCR2, IDO2*, *IL15*, *IL2RB*, *PDGFB*, and *S1PR1* were lowly expressed. More importantly, the most striking differences in GE were observed for *MRC1* (CD206), *MARCO*, *CCL3*, *CCL4*, *CCL18*, *CCL20*, *CXCL3*, *CXCL5*, *CXCL8*, *CD1B*, *CD38*, *CD40*, *CD74*, *CD274* (PD‐L1), *CD301* (CLEC10A), *CSFR2*, *CTSC*, *FLT1* (VEGFR), *ICAM1* (CD54), *IL10RA*, *SOCS3*, *STAT3*, *TNFSF13* (APRIL), and *VEGFA.* Moreover, *HLA‐class II* molecules were expressed higher in L428‐educated Mφ than in DLBCL‐CM and M‐CSF Mφ. GE of *PECAM1* (CD31), *CD226*, *and SPARC*, in contrast, did not differ between L428‐educated Mφ and M‐CSF Mφ, but was distinctly lower in DLBCL‐derived Mφ. On the other hand, GE of *ADAM9*, *MMP9*, *CD52*, *CD300C*, *CD300LB*, *CD302*, *CTSB*, *CTSD or PDGFRA and TNFRSF11A* (RANK) was higher in DLBCL‐CM‐treated Mφ than in L428‐educated Mφ. Therefore, it is proposed that L428‐educated Mφ have an M2‐like phenotype and differ from DLBCL‐CM and M‐CSF‐educated Mφ.

Next, a gene set enrichment analysis was performed. A KEGG pathway comparison between M‐CSF and HL‐CM‐educated Mφ revealed, for example, that Jak/STAT signaling and antigen presentation are more pronounced in L428‐CM‐treated Mφ (Fig. S2).

To gain deeper insight into the functions of the L428‐educated Mφ, label‐free data‐independent liquid chromatography–tandem mass spectrometry was used to determine protein expression levels in Mφ. Protein levels were then correlated with the corresponding transcript levels. This analysis confirmed, among others, the striking difference in (*MRC1*)/CD206 expression, which was significantly higher in L428‐educated Mφ than in both, M‐CSF and DLBCL‐CM‐derived Mφ (Table 1). Gene and protein expression data can be found in the Tables [Link], [Link], [Link], [Link], [Link].

**Figure 2:**
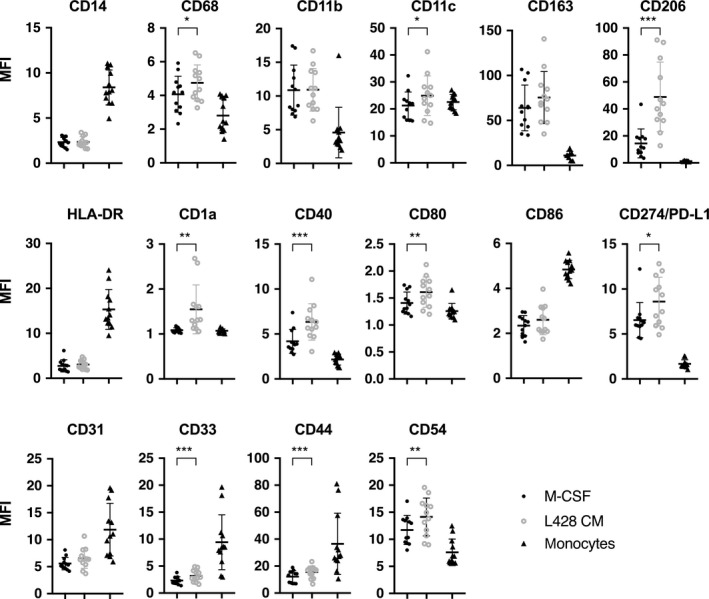
Surface expression of selected proteins on M‐CSF or L428‐CM‐educated Mφ and monocytes. Flow cytometry analysis of surface protein expression on monocytes and Mφ differentiated with either 2.5 ng·mL^−1^ M‐CSF or L428‐CM. MFI was calculated by dividing the MFI of the specific antibody by isotype MFI (mean ± SD, *n* = 12, paired *t*‐test, two‐tailed; *P < 0.05, **P < 0.01 and ***P < 0.001).

Next, we used flow cytometry to compare the expression of CD68, CD163, CD206, CD14, (HLA)‐DR, CD1a, CD11b, CD11c, CD40, CD80, CD86, CD274/PD‐L1, CD31, CD33, CD44, and CD54 between M‐CSF and L428‐educated Mφ and monocytes from 12 different donors (Fig. 2). In concordance with the omics data, CD206 was the most abundant molecule expressed on the surface of L428‐educated Mφ. We also observed higher expression of CD68, CD1a, CD80, PD‐L1, CD40, CD3, CD44, and CD54, further supporting the notion that L428‐CM‐derived Mφ represent an M2‐like subtype distinct from that of M‐CSF‐derived Mφ. Additional flow cytometry data can be found in Fig. S3. It is evident that CD206 is expressed more homogeneously on L428‐CM than M‐CSF educated Mφ. GE analysis of selected M1 (IL8/CXCL8, IL1ß, and CXCL9) and M2 (MRC1, CSF‐1, and CCL22) markers lend further support to the flow cytometry data. GE of Mφ stimulated with M‐CSF, LPS/IFNγ, IL4/IL13, IL10, L428‐CM and L1236‐CM, respectively, was compared to that of unstimulated monocytes. The GE profiles concord with the M1‐/M2‐profiles and further support the view that HL‐educated Mφ can be defined as M2‐like (Fig. S4).

**Figure 3:**
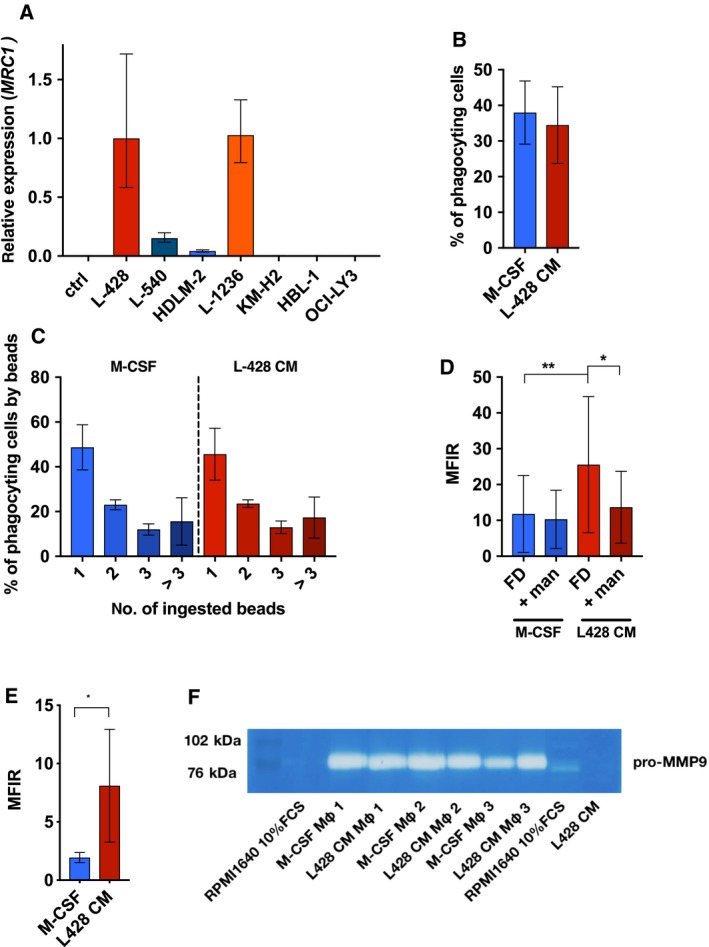
MRC1 gene expression, endocytotic activity, and type I collagen uptake by Mφ. (A) Gene expression of the MRC1 (CD206) in Mφ differentiated with either 2.5 ng·mL^−1^ M‐CSF (ctrl) or lymphoma‐derived CM as indicated. (B–D) Mφ were differentiated in Teflon‐coated cell culture bags with either 2.5 ng·mL^−1^ M‐CSF or L428‐CM for 7 days and transferred to cell culture dishes. After 3 h, nonadherent cells were washed off and adherent cells were incubated with (B/C) latex beads (5 beads/cell) for 2 h at 37 °C or on ice. Fluorescence was measured by flow cytometry. The overall percentage of phagocytic cells and their percentages as a function of the number of beads taken up were calculated by subtracting the respective percentages determined on ice from those at 37 °C (mean ± SD; *n* = 12). (D) Mφ were incubated with 1 mg·mL^−1^ 10‐kDa FITC dextran for 2 h at 37 °C and on ice. For blocking, mannose was given 10 min prior to dextran. Fluorescence was measured by flow cytometry. MFI ratios (MFIR) were calculated by dividing the MFI of 37 °C samples by the MFI of corresponding samples on ice (mean ± SD; *n* = 12, paired one‐way ANOVA with Bonferroni’s post‐test). (E) Mφ were incubated with 5 µg·mL^−1^ fluorescence‐labeled gelatin OG‐488 conjugate for 30 min at 37 °C or on ice. MFIR were calculated dividing the MFI of a 37 °C sample by the MFI the corresponding sample kept on ice (mean ± SD, *n* = 5, paired *t*‐test, two‐tailed; *P < 0.05, **P < 0.01). (F) MMP‐9 levels in Mφ differentiated by M‐CSF or L428‐CM treatment were analyzed by zymography. The cell‐free cell culture supernatants were diluted 1 : 40 before applying to the gelatin gel. Experiments using three different donors are shown.

In summary, the stimulation of monocytes with L428‐CM or M‐CSF changes the abundance of surface markers commonly used to monitor Mφ differentiation. This shows that after 7 days, mature Mφ are generated, which belong to the M2 subtype based on high CD163 and CD206 expression. Compared to M‐CSF‐educated Mφ, L428‐CM‐differentiated Mφ express more CD1a, CD80, CD40, and PD‐L1, which are involved in T‐cell interactions. The increased expression of CD11c, CD206, CD33, CD44, and CD54, on the other hand, points toward enhanced cell–matrix or cell–cell interactions. The other markers analyzed were not differentially expressed between L428‐CM and M‐CSF‐treated Mφ. Importantly, CD206 upregulation in lymphoma‐educated Mφ was also observed for L1236 and L540‐CM‐derived Mφ when compared with HBL1 and OCI‐Ly3‐CM (Fig. 3A). This further emphasizes that the ability of HL cells to differentiate monocytes into M2‐like Mφ, which might play important roles in T‐cell interactions as well as cell–matrix or cell–cell interactions involving the MRC1 (CD206; Taylor *et al.*, 2005).

### 3.2 HL‐educated macrophages are characterized by mannose‐dependent endocytosis and increased collagen ingestion

The strong upregulation of CD206 expression in L428‐CM vs. M‐CSF differentiated Mφ suggested that the former might differ in their endocytic capacity. Therefore, various endocytosis assays were performed. First carboxylate‐modified fluorescence‐labeled latex beads were used to monitor phagocytic activity (Fig. 3B,C). We observed no differences in the percentage of cells that took up latex beads or in the number of beads taken up, indicating that L428‐CM and M‐CSF‐differentiated Mφ do not differ in the uptake of large particles.

Since MRC1/CD206 can bind and take up polysaccharides, glycosylated proteins, and collagen (Kato *et al.*, 2000; Taylor *et al.*, 2005), we tested whether HL‐CM‐treated Mφ differed from M‐CSF‐differentiated Mφ in the uptake of polysaccharides and collagen. We incubated the respective Mφ with 10‐kDa FITC dextran in the presence or absence of mannose and measured the uptake of FITC dextran by flow cytometry (Fig. 3D). Mφ differentiated with L428‐CM took up significantly more dextran than M‐CSF‐derived Mφ. More importantly, addition of mannose led to a significant inhibition of the dextran uptake in L428 CM‐educated Mφ, whereas M‐CSF‐induced Mφ showed only mannose‐unrelated dextran uptake. This also applied to 70‐kDa FITC dextran indicating that the higher dextran uptake by HL‐CM‐educated Mφ is mediated by CD206 (data not shown). Next, we tested the uptake of type I collagen by adding fluorescence‐labeled gelatin (gelatin OG‐488) to Mφ (Fig. 3E). Interestingly, as observed for dextran, L428‐CM‐educated Mφ also showed a higher capacity of collagen uptake, further supporting the notion that CD206 on HL‐associated M2‐like Mφ may participate in the remodeling of the lymphoma microenvironment (Madsen *et al.*, 2013).

We also tested whether HL‐educated Mφ secrete the matrix metalloproteinases MMP2 and MMP9, which are involved in extracellular matrix (ECM) degradation. Zymography, using gelatin‐containing gels, failed to detect any activity of MMP2 in supernatants of HL‐ and M‐CSF‐induced Mφ, whereas both secreted equally large amounts of MMP9 (Fig. 3F). L428 cells, in comparison, secreted far less MMP9.

In conclusion, HL‐CM‐differentiated Mφ differ from M‐CSF‐induced Mφ in their ability to take up CD206‐specific targets. Further, MMP9 secretion hints at an active role of TAMs in remodeling the ECM.

### 3.3 IL13 is present in HL‐CM and upregulates CD206 in monocytes

**Figure 4:**
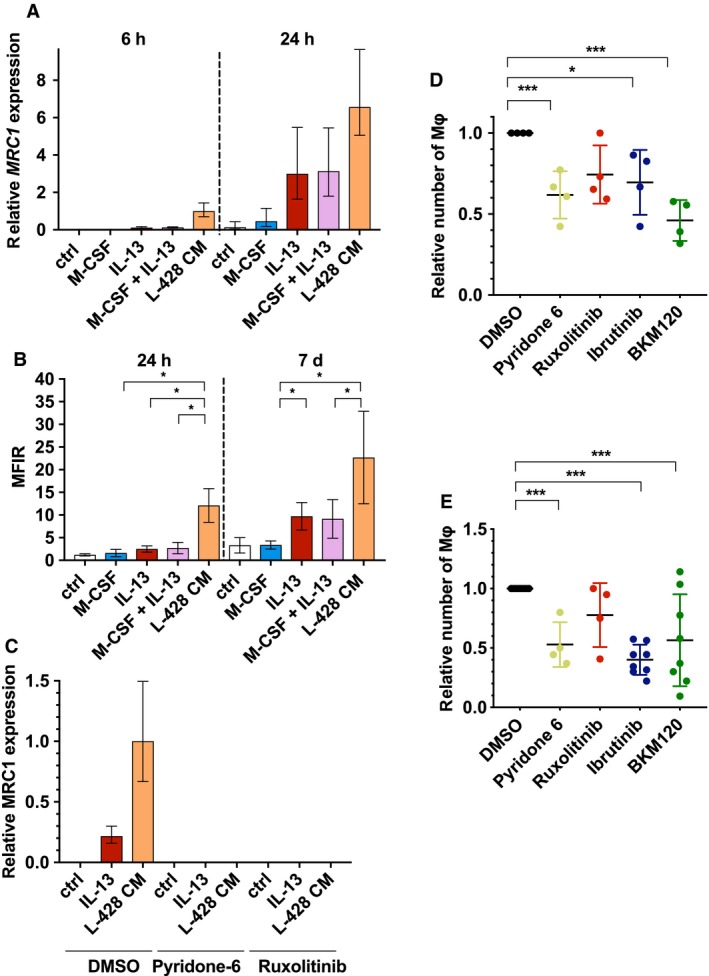
CD206/MRC1 is upregulated by IL13 and L428‐CM. (A) CD14^+^ PBMCs were stimulated with 2.5 ng·mL^−1^ M‐CSF, 10 ng·mL^−1^ IL‐13, or both, or with L‐428‐CM for the indicated time. Gene expression of *MRC1* was analyzed by qRT–PCR, relative to *GAPDH* (mean ± SD, *n* = 6). (B) Monocytes were seeded in Teflon‐coated cell culture bags with 2.5 ng·mL^−1^ M‐CSF, 10 ng·mL^−1^ IL‐13, or both, or with L‐428‐CM mixed with an equal volume of fresh medium. Aliquots of Mφ were taken at the indicated time points and stained with anti‐CD206 antibody. MFIRs were calculated as described above (mean ± SD, *n* = 6; paired one‐way ANOVA with Bonferroni’s post‐test; C) CD14^+^ PBMCs were preincubated with DMSO or inhibitors for 1 h before 10 ng·mL^−1^ IL‐13 or L‐428‐CM were added. Monocytes were cultured for six additional hours. Gene expression of *MRC1* was analyzed by qRT–PCR, relative to *GAPDH* and *MRC1* expression in L428‐CM‐treated monocytes (mean ± SD, *n* = 6). (D/E) Mφ were differentiated in Teflon‐coated cell culture bags with either 2.5 ng·mL^−1^ M‐CSF (D) or L428 CM (E) for 7 days in the presence of DMSO or indicated inhibitors (1 µm), and cells were counted (mean ± SD, *n* = 4/6; paired one‐way ANOVA with Bonferroni’s post‐test; *P < 0.05, and ***P < 0.001).

A distinguishing feature of HL cells is their ability to express and secrete IL13 and M‐CSF (Lamprecht *et al.*, 2010; Scott and Steidl, 2014; Skinnider and Mak, 2002; Tudor *et al.*, 2014). Additionally, Mφ are known to be polarized toward an M2 phenotype by IL4 or IL13, which includes upregulation of CD206. However, whether either or both IL13 and M‐CSF drive CD206 expression remained to be elucidated (Mantovani *et al.*, 2002). Therefore, we isolated CD14^+^ PBMCs and stimulated them with M‐CSF, IL‐13, or a combination of both in direct comparison with L428‐CM (Fig. 4A). In parallel, the amount of IL13 and M‐CSF secreted by HL cells used in this study was determined by ELISA. Both L428 and L1236 secreted large amounts of IL13 and M‐CSF, whereas DLBCL cells secreted neither (Fig. S5). We monitored *MRC1* GE after 6 and 24 h of stimulation. There was no detectable expression of *MRC1* in unstimulated and M‐CSF‐stimulated monocytes after 6 h. However, upon stimulation with either IL13 or L428‐CM, *MRC1* was expressed at 6 h, and even more so after 24 h. Addition of M‐CSF did not further increase IL13‐induced *MRC1* expression. Compared to IL13 only, L428‐CM‐induced expression of CD206/MRC1 was about tenfold higher after 6 h and remained twofold higher after 24 h. In addition, the surface expression of CD206 was analyzed after 24 h and 7 days (Fig. 4B). In concordance with *MRC1* GE levels, surface expression was increased in IL13 and L428‐CM‐treated cells after 24 h but not in untreated controls or M‐CSF‐stimulated cells. Again, L428 CM‐treated cells expressed about four times more CD206 than IL13‐treated cells. Its expression further increased after 7 days and was then also detectable on unstimulated and M‐CSF‐treated cells. This suggests the activation of an intrinsic program, which needs more time to support *MRC1* expression, and that HL‐derived IL13 promotes this process.

Having shown that IL13 was involved in the regulation of *MRC1* GE and CD206 protein abundance on the cell surface of Mφ, we tested the importance of Jak/STAT signaling. We monitored *MRC1* GE in monocytes after 24 h of stimulation with or without ruxolitinib, which targets Jak1 and 2, or pyridone 6, which inhibits all four Janus kinases (Fig. 4C; Thompson *et al.*, 2002; Verstovsek, 2013). Both IL13 and L428‐CM induced *MRC1* expression as shown above. The co‐incubation with either ruxolitinib or pyridone 6 completely abrogated *MRC1* gene activation by IL13 or L428‐CM. This argues for a Jak/STAT‐dependent regulation of *MRC1* expression. It also supports the presence of other Jak/STAT regulating factors in L428‐CM, in addition to IL13, that are responsible for an even higher upregulation of *MRC1* in L428‐CM‐educated Mφ. In parallel, an inhibitor screen was performed to estimate not only the impact of ruxolitinib or pyridone 6 on *MRC1* expression but also to test their impact on the differentiation of Mφ. We performed Mφ differentiation as described above for 7 days in Teflon‐coated cell culture bags either with M‐CSF or L428‐CM with and without ruxolitinib or pyridone 6, in comparison to Ibrutinib and BKM120 (Fig. 4D,E). The strongest effect was observed for Ibrutinib in L428‐CM‐educated Mφ. The pan‐Jak inhibitor pyridone 6 reduced Mφ differentiation to nearly 50%, whereas ruxolitinib seemed to affect only the outcome of M‐CSF‐differentiated Mφ. Both M‐CSF and L428‐CM‐induced Mφ differentiation was inhibited by the PI3K inhibitor BKM120. Our observation argues for the involvement of the tyrosine kinase BTK, PI3K and the Jak/STAT pathway in Mφ differentiation. It also indicates that IL13 and IL13‐activated Jak/STAT signaling, albeit important for *MRC1* expression and CD206 upregulation, is only partially involved in L428‐CM‐mediated TAM differentiation. Furthermore, our data imply that M‐CSF also activates the Jak/STAT and PI3K pathways, with differentiation being dependent to some extent on BTK. These similarities and differences between M‐CSF and HL‐educated Mφ need to be further dissected in the future.

### 3.4 Lymphoma–Mφ interactions in a preclinical model

**Figure 5:**
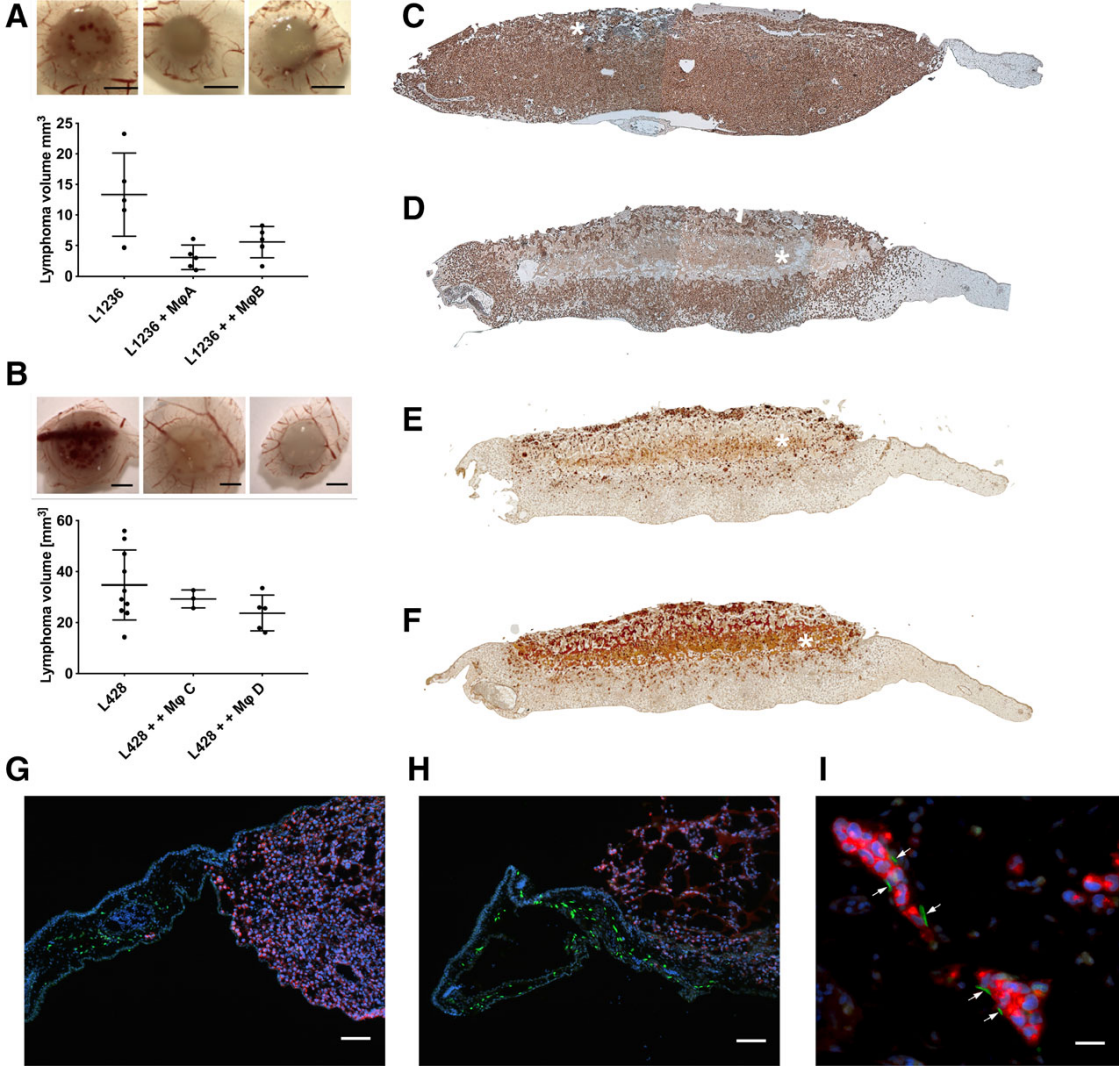
Mφ affect lymphoma growth in the chick chorioallantoic membrane (CAM). (A/B) Tumor volumes are reduced in the presence of Mφ. L1236 and L428 lymphomas were mixed with Mφ, inoculated on the CAM, and harvested after 4 days of tumor growth. To evaluate the lymphoma outcome, tumor volumes were defined using micro‐CT. Above the graphs are representative macroscopic images (with 7.8× magnification). CAM lymphomas (A) and respective CAM‐Mφ‐lymphomas (B) are shown. PBMCs from four different donors were used (Mφ A, B, C, D). Note the absence of bleedings in lymphomas with Mφ (mean ± SD; scale bar represents 2 mm). (C) Tissue section of L428 lymphoma stained for CD30. (D) Tissue section of L428 lymphoma with Mφ stained for CD30. (E) Tissue section of L428 lymphoma with Mφ stained for CD68. (F) Tissue section of L428 lymphoma with Mφ stained for CD206. Co‐expression of both CD68 and CD206 on Mφ shows that the M2‐like phenotype is maintained. (C–F) Note some remaining Matrigel (white asterisk) in L428 lymphomas with Mφ. Both, lymphoma cells and Mφ collectively invade the CAM. However, Mφ seem to consist of two populations, one migrating with the lymphoma cells and the other remaining at the site of application. See also Fig. S6D,E. (G) Staining of cryosections of CAM lymphoma of L428 cells and (H) L428 cells with L428‐CM‐educated Mφ with anti‐Prox1 (green) and anti‐CD30 (red) to visualize lymphatic vessels and L428 HL cells. Scale bar: 160 µm. (I) Visualization of HL cells [CD30 (red)] within lymphatic vessels [Prox1 (green), white arrows] to demonstrate their lymphogenic dissemination. Scale bar: 40 µm. See also Fig. S7.

To test the interaction of lymphoma cells with monocytes or Mφ in an *in vivo* setting, we used the CAM assay as it recapitulates important aspects of human lymphoma as shown recently (Klingenberg *et al.*, 2014a, 2014b). One million Mφ derived by incubation of monocytes with HL‐CM were mixed with 2 × 10^6^ lymphoma cells (L428, L1236) in Matrigel and applied on the CAM. Four days after application, lymphomas were harvested and characterized macroscopically and histologically by defining their volume and investigating the topography of lymphoma cells and Mφ in relation to lymphatics and blood vessels (Fig. 5).

The first observation concerned the reduced size of hemorrhages in lymphomas with Mφ (Figs 5A,B and S6A). This was confirmed by micro‐CT using PTA to enhance the contrast of blood (Cunningham and Crane, 1966; Saccomano *et al.*, 2018). This may also explain in part the smaller size of lymphomas co‐inoculated with Mφ compared to lymphomas without Mφ (Figs 5A,B and S6B). Moreover, immunostaining with α‐CD30 revealed that HL‐derived CAM lymphomas have a more compact morphology, with lymphoma cells invading the CAM and occupying the whole area between the upper chorionic and lower allantoic epithelium (Figs 5C and S6C). Staining CAM‐Mφ‐lymphoma specimens with antibodies against CD30, CD68, and CD206 revealed a compartmentalization of the tumor (Figs 5D–F and S6D). CD30‐positive HL cells were located in the upper and lower parts of the tumor and were almost absent in the center. CD68− and CD206‐positive Mφ dominated the upper and central parts and were present both in the remaining Matrigel and intermingled with tumor cells that had invaded the CAM. Comparable results were observed when applying CD14^+^ PBMCs together with L428 cells on the CAM, further supporting the observation that HL cells support the differentiation of monocytes to M2‐like Mφ with high CD206 expression (Fig. S6E).

A detailed immunofluorescence analysis of consecutive sections using costaining against CD30 or CD68 with Prox1, a marker for lymphatic endothelial cells, revealed the absence of lymphatic vessels in L428 CAM lymphomas (Fig. 5G). In CAM‐Mφ‐lymphomas, lymphatic vessels were regularly observed at the invasive front (Figs 5H and S7). Dissemination of lymphoma cells in lymphatics was regularly visible in CAM‐Mφ‐lymphomas (Figs 5I and S7).

### 3.5 CD206 and primary Hodgkin lymphoma cases

**Figure 6:**
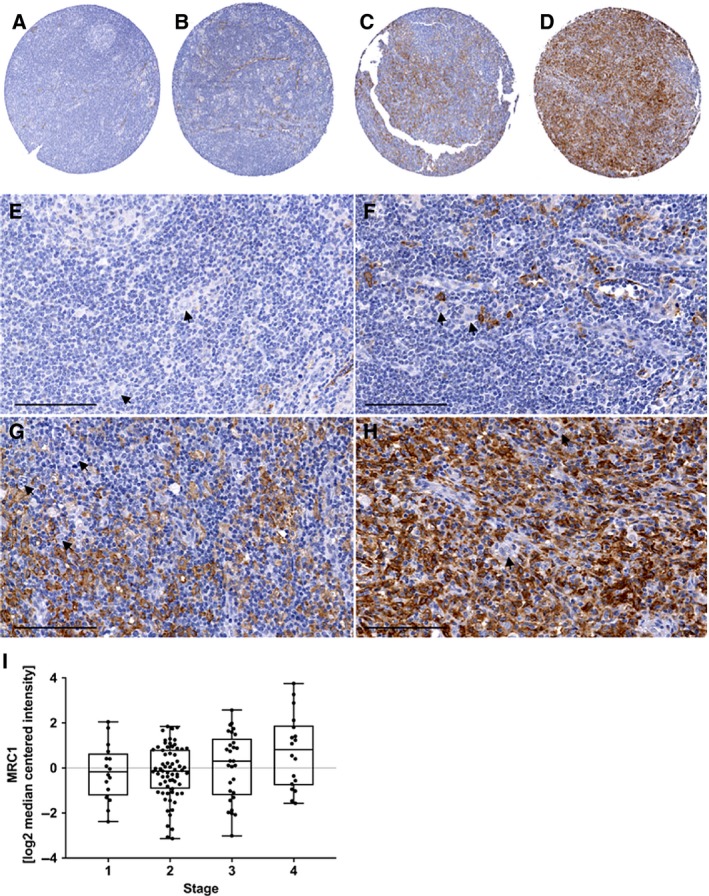
Different patterns of CD206‐positive cells in classical HL, and gene expression of *MRC1* (CD206) in cHL patients of different stages. (A–D) Four examples of a TMA with a total of 16 specimens demonstrating variable staining patterns for CD206 in classical HL. (E–H) Details of figures A–D. (A, E) Very few positive cells; (B,F) small number of positive cells; (C,G) moderate number of positive cells; (D,H) high number of positive cells. Scale bar in E‐H = 100 µm. Arrows show Hodgkin and Reed–Sternberg cells, which are negative for CD206 but often in close contact with Mφ. (I) MRC1/CD206 expression data obtained by Steidl et al. were correlated with the stage of cHL patients. A significant linear trend for high CD206 expression for cHL patients with stage 4 is described (ordinary one‐way ANOVA, slope 0.3219; *P* = 0.0170; Steidl *et al.*, 2010).

Immunohistochemical analysis of a tissue microarray of 16 HL specimens showed that a few cases to feature a large count of CD206‐positive cells in the lymphoma tissue (Fig. 6A–H). Additional immunofluorescence analysis revealed that while CD163 and CD206‐positive Mφ are rare in the germinal center of reactive tonsils, they are readily detected in HL tissues, including CD163/CD206 double‐positive Mφ (Fig. S8).

Analysis of publicly available patient microarray data revealed a significant and linear trend for high CD206 expression in HL patients with stage IV (Fig. 6I). This supports the view that Mφ with high CD206 and, thus, probably increased matrix‐remodeling capacity are characteristic for advanced stages of classical HL.

## 4 Discussion

The lymphoma microenvironment plays an important role in the pathogenesis of HL. TAMs are a regular element of this microenvironment. Hence, it is essential to understand the mechanisms that enable HL cells to recruit and reprogram monocytes into TAMs (Aldinucci *et al.*, 2010; Steidl *et al.*, 2011). However, despite the negative impact of increased counts of M2‐like Mφ on outcome and treatment response in HL, there is still more to be learnt about the mechanisms driving the development of HL‐associated Mφ (Scott and Steidl, 2014). Our data provide a mechanistic insight into the processes that are involved in the interaction of HL cells with Mφ. We present a combined analysis of transcriptome and proteome data to compare lymphoma‐educated to M‐CSF‐differentiated Mφ. We extend recent data and models, which have proposed CXCL13, CCL5, and M‐CSF as central players in the interaction of HL cells with Mφ (Carey *et al.*, 2017; Casagrande *et al.*, 2019; Ford *et al.*, 2015; Locatelli *et al.*, 2018; Ruella *et al.*, 2017; Scott and Steidl, 2014; Tudor *et al.*, 2014). We demonstrate that Jak/STAT signaling and, especially, IL13 are involved in the regulation of the expression of *MRC1* and the abundance of CD206 on the surface of HL‐educated Mφ. Thus, our study expands the role of IL13 from an autocrine growth factor to a lymphoma‐secreted factor that mediates ECM remodeling and immunosuppression by HL‐educated Mφ (Skinnider and Mak, 2002), which in turn facilitates the dissemination of lymphoma cells.

We observed significantly higher counts of Mφ upon differentiation with HL‐CM as compared to DLBCL‐CM or M‐CSF. Although M‐CSF is expressed by the majority of HL cell lines, and M‐CSF is known to promote Mφ differentiation into M2‐like subtypes, the observed phenotype and the strongly enhanced CD206 surface expression cannot be attributed to M‐CSF in HL‐CM. We observed that IL13 is involved in early *MRC1* mRNA upregulation and enhanced CD206 incorporation into the cell membrane. Enhanced IL13 expression is characteristic of HL, and it has been shown that IL13 can promote Mφ differentiation (McKenzie *et al.*, 1993; Skinnider *et al.*, 2001). Conditioned media of L428 and L1236 cells, which featured the highest capacity to attract monocytes and Mφ, were also characterized by a high potential to activate *MRC1* GE in Mφ. Both HL cell lines secreted large amounts of M‐CSF and IL13. The same was true for HDLM2 cells (Fig. S5), but in contrast to L428 and L1236 cells, they failed to induce *MRC1* GE in Mφ (Fig. 3A) for reasons yet to be elucidated. However, the observation hints at other not yet identified Jak/STAT activating factors that complement IL13. The latter binds to an IL4Rα‐IL13Rα heterodimer, which intracellularly can interact with JAK1, JAK2, and TYK2 (Bhattacharjee *et al.*, 2013). The absence of *MRC1* expression in Mφ after ruxolitinib treatment indicates that either JAK1 or JAK2 rather than TYK2 induces M2‐like GE. Thus, IL13 seems to be not only involved in the autocrine support of HL cells but also in rebuilding the lymphoma microenvironment through Mφ differentiation and upregulation of CD206. This offers novel opportunities for targeting the lymphoma microenvironment by Jak/STAT inhibition (Kim *et al.*, 2014). Whether this approach can be used in combination or extension of immune checkpoint inhibitor therapies needs to be evaluated in future studies.

Preliminary data show the additional involvement of BTK‐ and PI3K‐regulated pathways, as respective inhibitors affect both M‐CSF‐ and L428‐CM‐mediated Mφ differentiation. This supports the recent observation of a preclinical model that used RP6530 to target the interaction between HL cells and Mφ (Locatelli *et al.*, 2018). We propose that M‐CSF induces a network of signaling pathways to activate autocrine Mφ differentiation factors, whereas IL13 directly promotes an early differentiation program. Although elevated M‐CSF serum levels have been reported in HL patients, and HL cells were found to be M‐CSF‐positive by immunohistochemistry, the measured M‐CSF amounts in the lymphoma‐CM *in vitro* and the differences in Mφ counts and phenotype indicate the involvement of additional factors during HL‐specific Mφ differentiation (Casagrande *et al.*, 2019; Kowalska *et al.*, 2012; Zheng *et al.*, 1999). Recently, an autoregulatory loop between TAMs and breast cancer cells has been reported (Cassetta *et al.*, 2019). It might be interesting to test whether described factors such as tumor necrosis factor alpha, SIGLEC1 and CCL8, which is self‐reinforcing through the production of M‐CSF, also participate in the interaction of HL cells with Mφ.

CD206 is a carbohydrate binding receptor expressed by Mφ, dendritic cells, and lymphatic endothelial cells. It belongs to a family of endocytic receptors with conserved structural elements consisting of an N‐terminal cysteine‐rich (CR) domain, a fibronectin type II (FNII) domain, and several C‐type lectin‐like domains (CTLDs; Taylor *et al.*, 2005; Wilting *et al.*, 2009). Through the CTLDs, CD206 can bind to glycoconjugates carrying terminal mannose, *N*‐acetylglucosamine, and fucose residues, while the FNII and CR domain bind to collagens and sulfated carbohydrates, respectively. CD206 is a highly effective endocytic receptor that constantly recycles between the plasma membrane and the early endosomal compartment. Various exogenous and endogenous ligands have been described for C‐type lectins, implicating several functions in homeostasis and inflammation. We observed that HL‐CM‐educated Mφ were able to take up collagen, which concords with previous reports on M2‐like Mφ (Madsen *et al.*, 2013). This suggests that these M2‐like Mφ play a specific CD206‐mediated role in lymphoma tissue reorganization.

Matrix remodeling is a common process in tumor progression, and the tumor stroma is characterized by profound proteolytic degradation (Cox and Erler, 2011; Nakayama *et al.*, 2016; Tataroglu *et al.*, 2007). The most striking feature of the nodular sclerosis subtype of classical HL (NSCHL) is the presence of ECM deposits. These are collagen‐rich fibrotic bands surrounding nodules of inflammatory and neoplastic cells. Of note, L428 cells are derived from NSCHL. The observed capacity of L428‐CM to induce a specific Mφ subtype with high CD206 expression and collagen uptake supports the hypothesis of tissue remodeling by the interaction of lymphoma cells with stromal cells, and adds an additional element to the previously observed mast cell infiltration and fibrosis in HL (Nakayama *et al.*, 2016). It has to be mentioned that C‐type lectins facilitate the endocytosis of particular antigen types for cross‐presentation, but whether antigen uptake by the C‐type lectins will result in immunogenic or tolerogenic cytotoxic T‐cell priming remains to be studied (Allen and Rückerl, 2017). Taking into account the capacity of HL‐CM to enhance CD40 and PD‐L1 cell surface expression on Mφ, and the recently described close association of HL cells with PD‐L1‐positive Mφ, it is reasonable to assume that HL‐educated Mφ modify T‐cell functions at different levels (Carey *et al.*, 2017).

In summary, we hypothesize that the observed *in vitro* and *in ovo* regulation of CD206 in Mφ might also occur *in vivo* and contribute to CD206 abundance in advanced stages of HL. Our data identify CD206 as a potential target for the modulation of Mφ activation in addition to the recently discussed targeting of the CSF1R (Luo *et al.*, 2006; Martín‐Moreno *et al.*, 2015; Pham *et al.*, 2018; Pyonteck *et al.*, 2013; Ries *et al.*, 2014; Wang *et al.*, 2018; Zhu *et al.*, 2014). Inhibition of the Jak/STAT pathway targets not only lymphoma cells directly, but may also affect lymphoma‐educated Mφ development and/or activity. This supports the simultaneous targeting of both lymphoma cells and Mφ with PI3K inhibitors, and the use of Maraviroc for the blocking of CCR5 in these cells (Casagrande *et al.*, 2019; Locatelli *et al.*, 2018). In line with this, targeting the pattern recognition receptor MARCO can alter Mφ polarization and result in reduced tumor growth (Georgoudaki *et al.*, 2016). This type of reprogramming may now be tested in HL, as HL‐educated Mφ show specific high MARCO expression. Furthermore, imaging of TAMs seems to be a valuable approach and mannose‐coated liposomes or nanobodies are tested for their use in monitoring CD206 TAMs using PET (Azad and Schlesinger, 2015; Locke *et al.*, 2012; Movahedi *et al.*, 2012).

## 5 Conclusion

The present study further corroborates the shown capacity of HL cells to attract and differentiate monocytes into alternatively activated M2‐like Mφ. The latter are characterized by high CD206 expression, which indicates that HL‐TAMs act as immunosuppressive agents. Further, CD206 is associated with a high capacity to bind and take up glycoconjugates with terminal mannose residues as well as type I collagen. Thus, it is likely that CD206 contributes to the remodeling of the tumor microenvironment. Our study highlights the role of Jak/STAT signaling and, particularly, the capacity of IL13 to upregulate CD206 expression on Mφ, thus expanding its role from an autocrine growth factor in HL to a lymphoma‐secreted factor capable of modulating the tumor microenvironment. Additionally, the increased expression of CD40 and PD‐L1 argues for a role of HL‐secreted factors in the reprogramming of monocytes/ Mφ to further support the capacity of HL cells to suppress, for instance, T‐cell‐mediated anti‐lymphoma responses. The CAM assay presents a simple preclinical model to study HL cell–Mφ interactions and the dissemination of HL cells. Finally, MRC1/CD206 expression data show a positive correlation with the stage of HL patients.

## Conflict of interest

The authors declare no conflict of interest.

## Author contributions

AA and FvB performed most of the experiments. AA, FL, and FvB conducted *in vitro* analyses of Mφ and cell lines, while AA, FvB, RSp, MM, MV, CD, GB, and JW contributed to specific experiments including flow cytometry, micro‐CT analysis of the chick chorioallantoic assay, and data interpretation as well as chick chorioallantoic model analysis. KL, WG, KD, and PO were involved in proteomic analysis and data interpretation; SRe and WK performed IHC analysis. JCE, PP‐R, TR, and RSPJ analyzed GE data. VB, JW, TB, FA, and LT were involved in manuscript writing and the final approval. AA, FL, TP, RS, JCE, and DK designed the research, analyzed, and interpreted data, RS, PJO, JW, and DK wrote the finally approved manuscript.

## Supporting information


**Fig. S1** **.** Hodgkin lymphoma cells secret factors that attract and differentiate monocytes into macrophages.
**Fig. S2** **.** KEGG pathway comparison between M‐CSF and HL‐CM‐educated Mφ.
**Fig. S3** **.** Flow cytometry analysis of surface expression of CD206, PD‐L1, and CD163 on L428‐CM and M‐CSF educated Mφ.
**Fig. S4** **.** Examples of gene expression of selected M1‐ and M2‐ Mφ markers.
**Fig. S5** **.** Amounts of IL13 and M‐CSF secreted by HL cells.
**Fig. S6** **.** Mφ affect lymphoma growth in the chick chorioallantoic membrane (CAM), blood vessel destruction and tissue remodeling.
**Fig. S7** **.** Dissemination of lymphoma cells in lymphatics in CAM‐Mφ‐lymphomas.
**Fig. S8** **.** CD163 and CD206 double staining in a representative tonsil and HL tissue sections.

**Table S1** **.** Differential gene expression of macrophages comparing HL‐CM and M‐CSF differentiated macrophages.

**Table S2** **.** Differential gene expression of macrophages comparing HL‐CM and DLBCL‐CM differentiated macrophages.

**Table S3** **.** Differential protein abundance of macrophages comparing HL‐CM and M‐CSF differentiated macrophages.

**Table S4** **.** Differential protein abundance of macrophages comparing HL‐CM and DLBCL‐CM differentiated macrophages.

**Table S5** **.** Differential protein abundance of macrophages comparing DLBCL‐CM and M‐CSF differentiated macrophages.
