# Supplementary material for: High CD206 levels in Hodgkin lymphoma‐educated macrophages are linked to matrix‐remodeling and lymphoma dissemination
Source: Mol Oncol. 2020 Jan 28;14(3):571–89. doi: 10.1002/1878-0261.12616 (PMC7053241; doi:10.1002/1878-0261.12616)
Supplement: Supplementary file 1 — Fig. S1 . Hodgkin lymphoma cells secret factors that attract and differentiate monocytes into macrophages. Fig. S2 . KEGG pathway comparison between M‐CSF and HL‐CM‐educated Mφ. Fig. S3 . Flow cytometry analysis of surface expression of CD206, PD‐L1, and CD163 on L428‐CM and M‐CSF educated Mφ. Fig. S4 . Examples of gene expression of selected M1‐ and M2‐ Mφ markers. Fig. S5 . Amounts of IL13 and M‐CSF secreted by HL cells. Fig. S6 . Mφ affect lymphoma growth in the chick chorioallantoic membrane (CAM), blood vessel destruction and tissue remodeling. Fig. S7 . Dissemination of lymphoma cells in lymphatics in CAM‐Mφ‐lymphomas. Fig. S8 . CD163 and CD206 double staining in a representative tonsil and HL tissue sections. [file MOL2-14-571-s001.docx]

**Supplemental Figures for**

A. Arlt et al. Hodgkin lymphoma-educated macrophages: high CD206 levels linked to matrix-remodeling and lymphoma dissemination

**Supplemental Figure 1: Hodgkin lymphoma cells secret factors that attract and differentiate monocytes into macrophages.** (**A**) Migration of monocytes in a Boyden chamber assay with a 5-µm porous membrane for 2 h toward 10% FCS or lymphoma CM (means ± SD, n = 10, one-way ANOVA with Bonferroni’s post-hoc-test). (**B**) 2 x10^6^ monocytes each were added to 6‑well Teflon culture dishes containing either medium supplemented with 2.5 ng/ml M‑CSF or in lymphoma CM mixed in equal parts with fresh medium. Cells were incubated for 7 d and afterwards macrophages were counted based on morphology and size (mean ± SD, n = 6, paired one‑way ANOVA with Bonferroni’s post‑hoc-test).

**
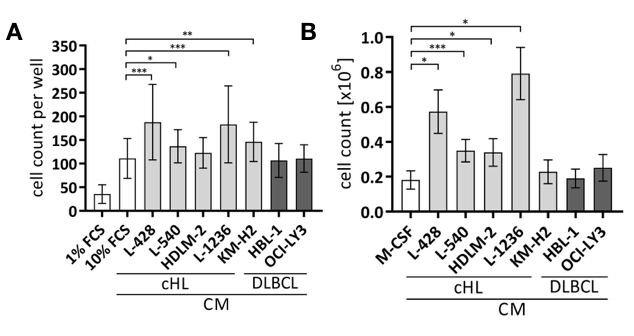
**

**Supplemental Figure 2:** KEGG-pathway comparison between M-CSF and HL-CM-educated Mφ. Graphical view for corresponding enrichments in the hematopoietic cell lineage, Jak/STAT signaling, NOD-like receptor signaling, antigen processing and presentation, and chemokine signaling. Green indicates reduced expression, red increased expression of corresponding pathway elements in L428 educated Mφ. The graphical presentation is based on Pathview and GAGE (1,2).

**
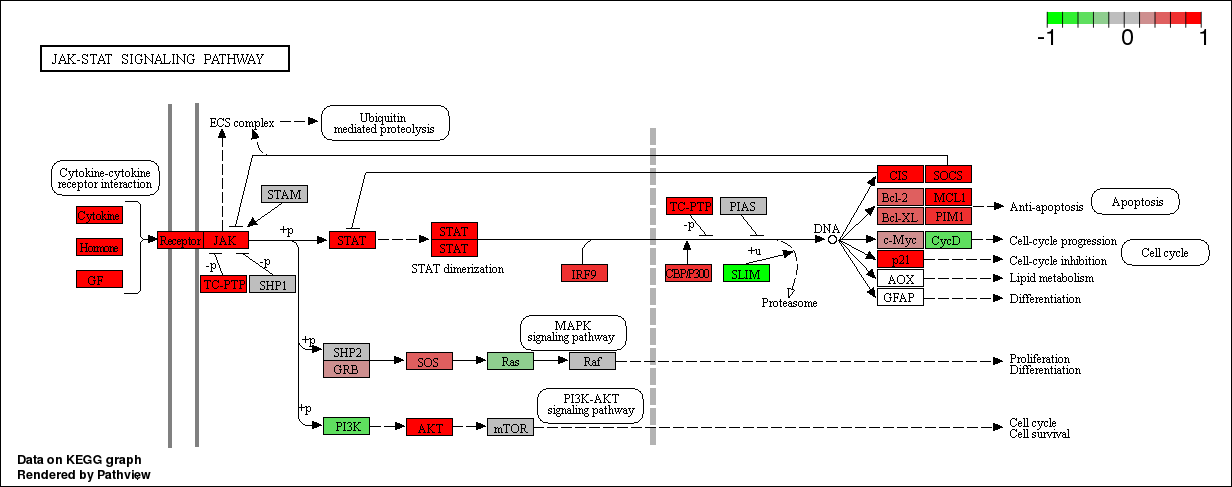
**

**
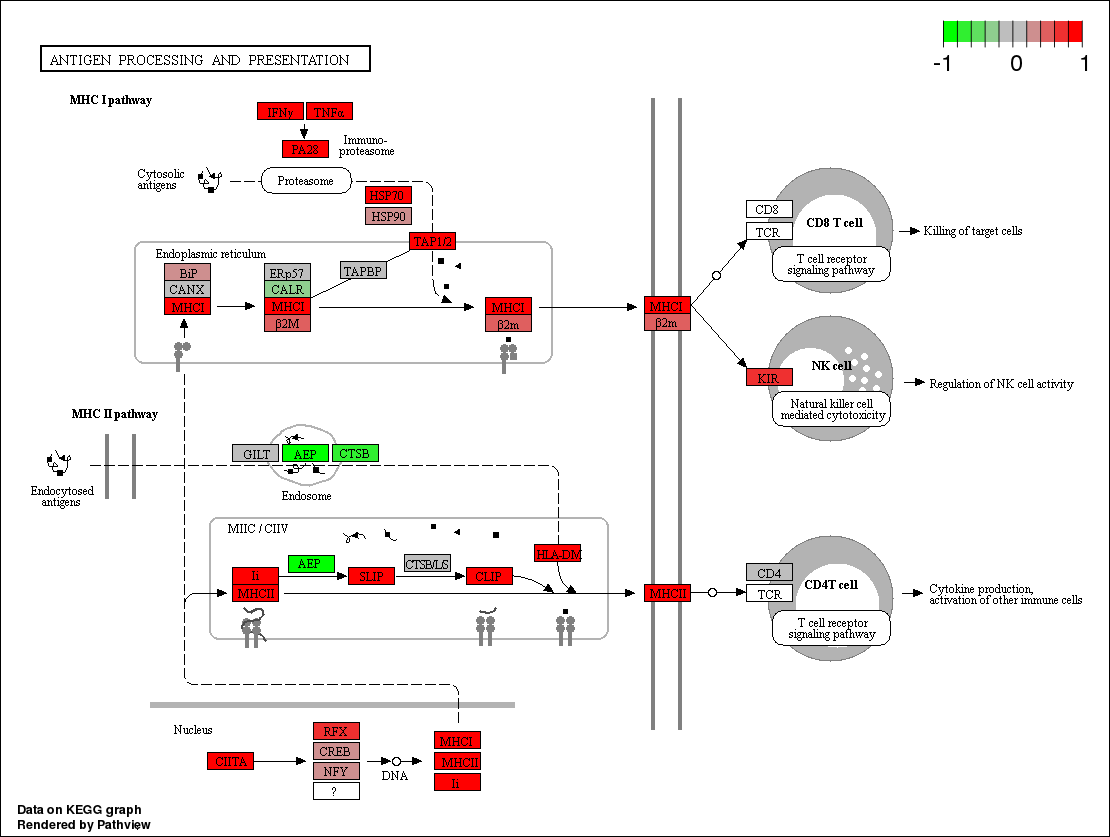
**

**
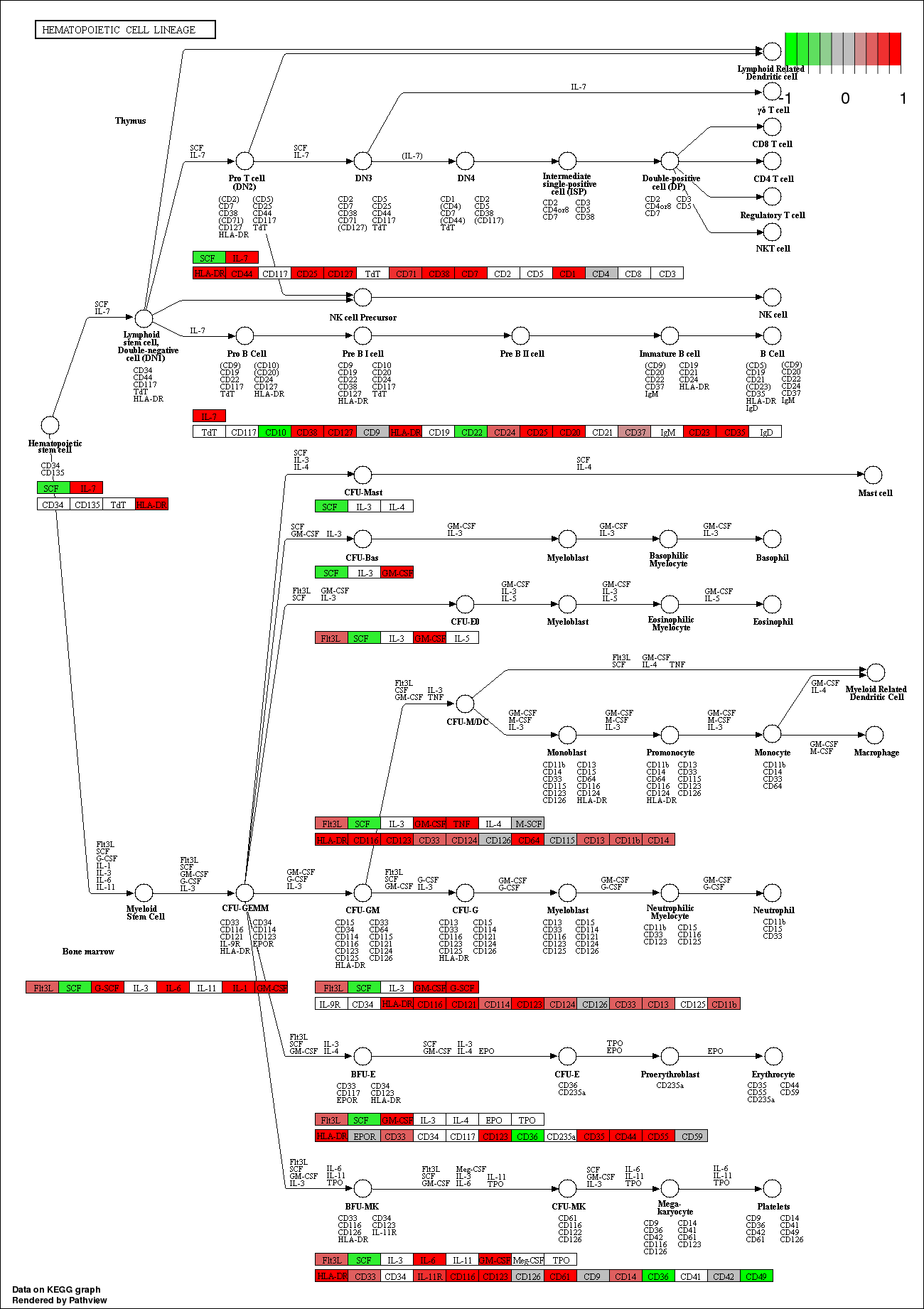

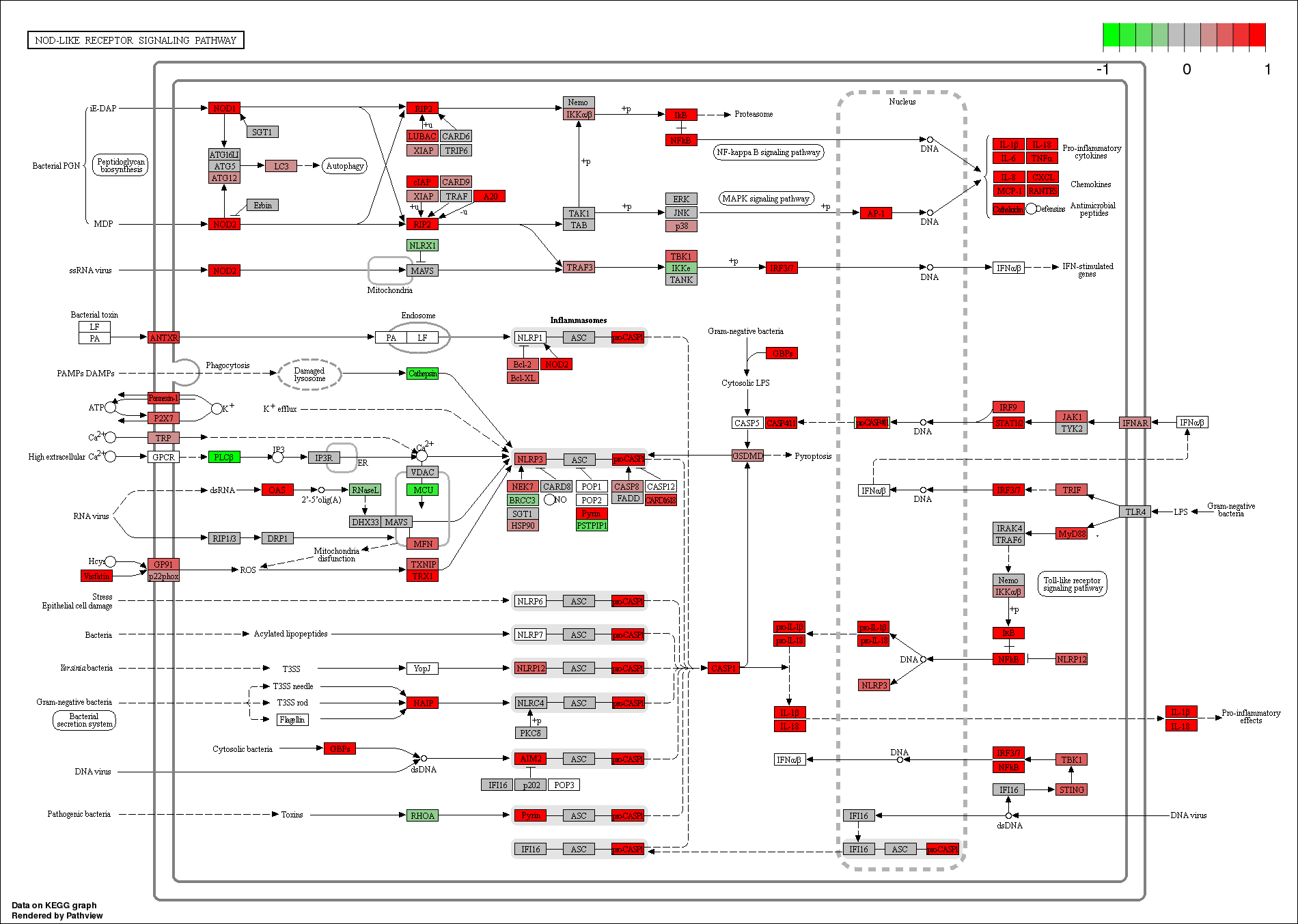

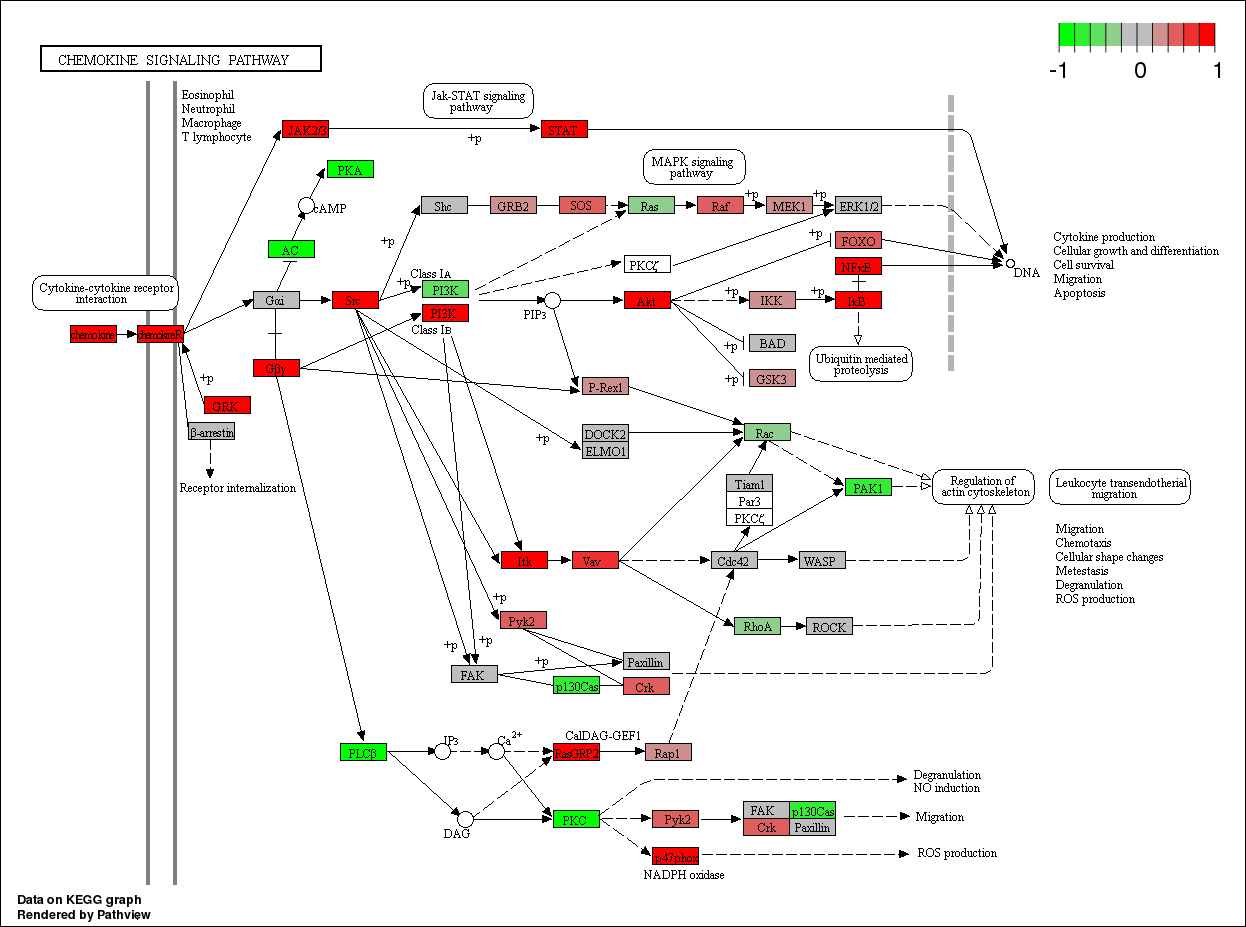
**

**Supplemental Figure 3. Flow cytometry analysis of surface expression of CD206, PD-L1, and CD163 on L428-CM and M-CSF educated Mφ**. Shown are the dot plots for six independent donors.

**Supplemental Figure 4: Examples of gene expression of selected M1- and M2- Mφ markers.** MRC1 (CD206) (A), CSF-1 (B) and CCL22 (C) gene expression were chosen as M2- Mφ markers, while IL8/CXCL8 (D), IL1ß (E) and CXCL9 (F) served as M1- Mφ markers. Mφ had been stimulated with M-CSF, LPS+IFN-γ or IL4+IL13, IL10, L428-CM and L1236-CM respectively. Expression was calculated relative to *GAPDH* and compared to an unstimulated control. Two independent donors were analysed.


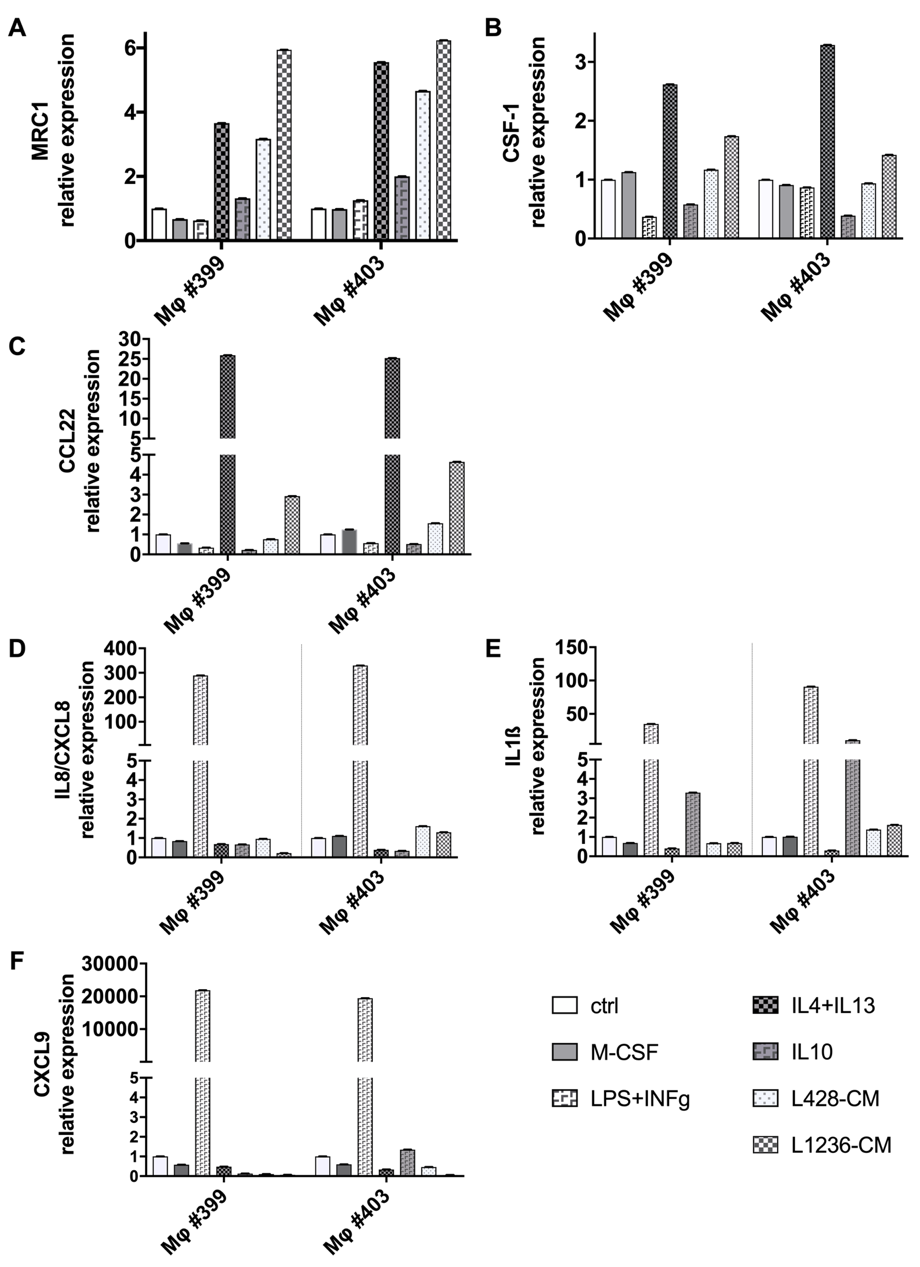


**Supplemental Figure 5:** **Amounts of IL13 and M-CSF secreted by HL cells.** 5x10^5^ cells/ml were cultured for 48 hrs, supernatant was harvested and the concentrations of (A) IL13 and (B) M-CSF were measured by ELISA according to the manufacturer’s instructions.

**
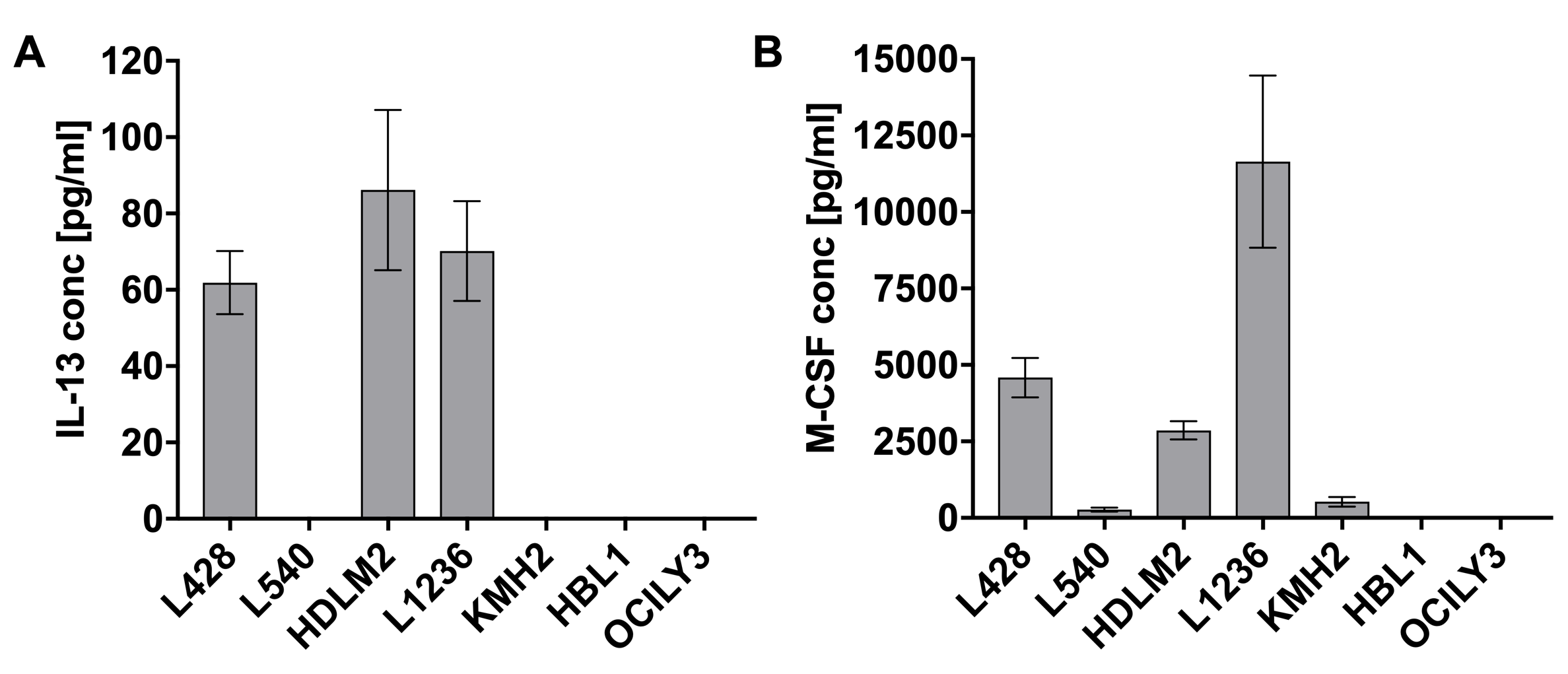
**

**Supplemental Figure 6: Mφ affect lymphoma growth in the chick chorioallantoic membrane (CAM), blood vessel destruction and tissue remodeling.**


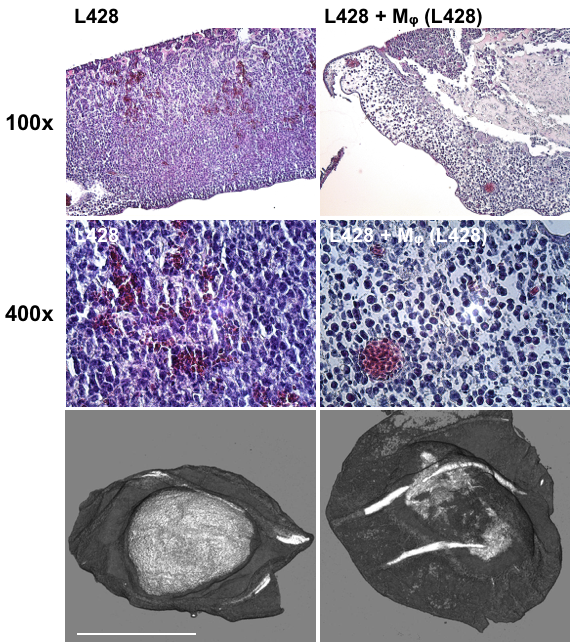


(**6A**) Comparison of HE-stained tissue sections of CAM lymphomas inoculated with L428 cells only or a mixture of L428 cells and L428-CM-educated Mφ. L428 only lymphomas show blood vessel destruction with hemorrhages, while L428 + Mφ lymphomas are characterized by loosely arranged tumor tissue. Micro-CT-pictures show different amounts of blood within CAM-lymphomas. The silver-grey areas represent hemorrhagic areas, supporting the observation of blood vessel destruction visualized on HE-stained tissue sections. This is based on the preferential staining of the erythrocyte cell membrane with phosphotyngstic acid, leading to a strong contrast of blood in micro-CT examinations (3,4). The scale bar in micro-CT pictures represents 5 mm.


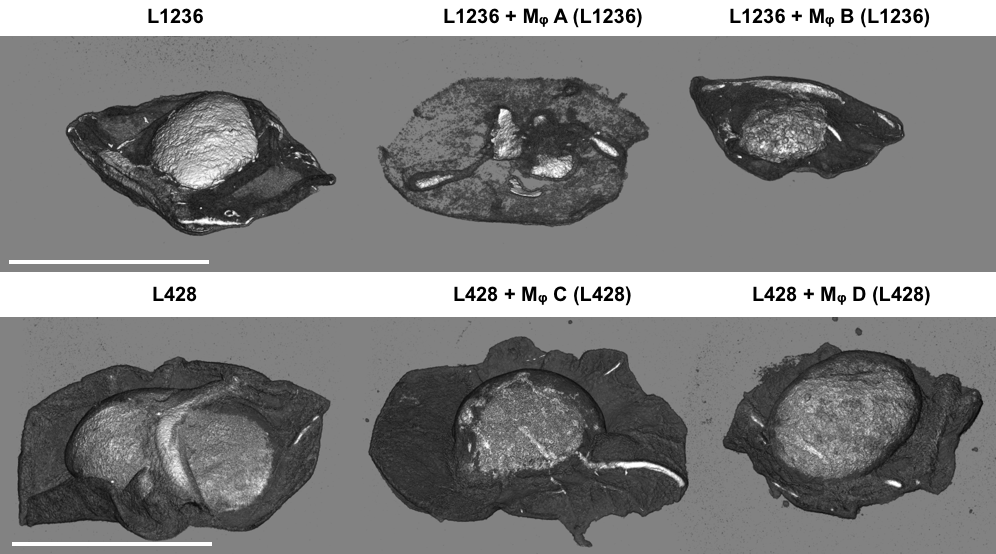


(**6B**) Examples of µCT-pictures of L1236 and L428 CAM lymphomas, respectively, without or with L1236/L428-CM-educated Mφ. Lymphoma volume was determined as in Figure 5A/B. The scale bar represents 6 mm.


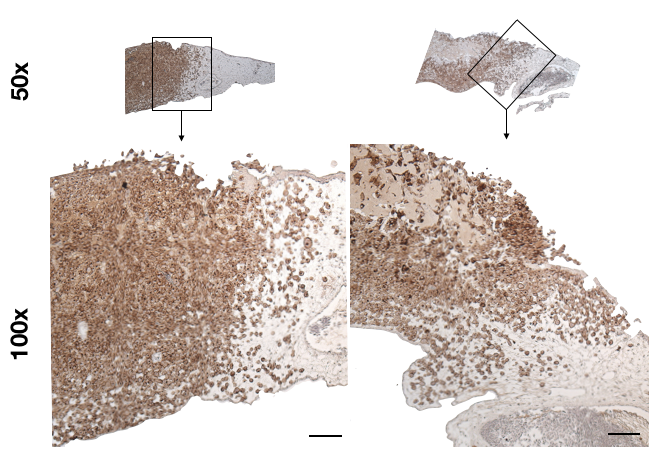


**(6C**) Comparison of anti-CD30 stained tissue sections of CAM lymphomas of L428 cells (left) and L428 cells co-inoculated with L428-CM-educated Mφ (right), demonstrating migration zones of lymphoma cells. The scale bar represents 120µm.


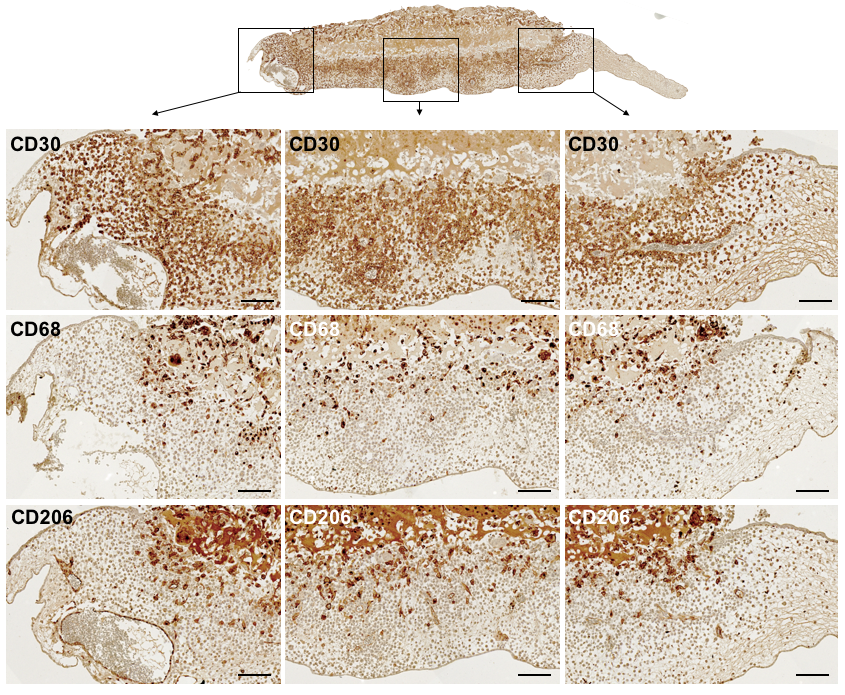


(**6D**) Tissue sections of L428 lymphoma with Mφ stained for CD30, CD68 and CD206, indicating expression of both CD68 and CD206, migration of Mφ out of the application zone (left panel), and migration with lymphoma cells towards blood vessels (right panel). The central panel shows the localization of tumor cells and Mφ in the center of the developed lymphoma. Please note that endothelial cells within the CAM are stained with α-CD206 antibodies (best visible in left panel). The staining of CD68 and CD206 allows to discriminate Mφ from endothelial cells. Magnifications are as in Supplemental Figure 6C. Scale bar represents 180µm.


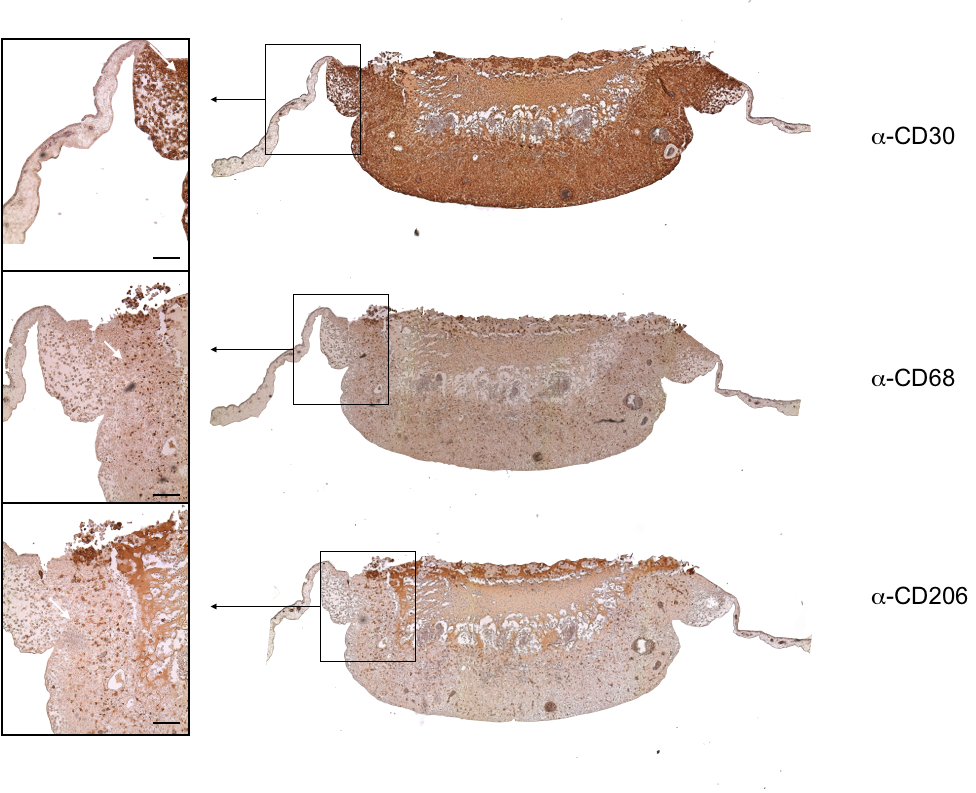


(**6E**) Tissue sections of L428 lymphoma with CD14+ PBMCs stained for CD30, CD68, and CD206. White arrows in the left panels show corresponding CD30, CD68 and CD206-positive cells, supporting the view that in the CAM co-culture model L428 cells are capable of promoting CD206 upregulation without prior *in vitro* Mφ differentiation. Magnifications are as in Supplemental Figure 6C. The scale bar represents 180µm.

**Supplemental Figure 7: Dissemination of lymphoma cells in lymphatics in CAM-Mφ-lymphomas.**

Staining of cryo**-**sections of a CAM lymphoma of L428 cells co-inoculated with L428-CM-educated Mφ with α-Prox1 (green) and α-CD30 (red) to visualize lymphatic vessels and L428 HL cells, respectively, and α-CD68 (red) to stain for Mφ. Tissue sections are stained with DAPI to visualize nuclei. (A/B) serial sections of CAM-lymphoma tissue sections stained with α-Prox1/α-CD30 (A) or α-Prox1/α-CD68 (B). Tumor cells and Mφ are migrating towards a blood vessel with chicken erythrocytes inside. (C/D) serial sections of CAM-lymphoma tissue sections at the border between the invading lymphoma cells with Mφ and the normal CAM stained with α-Prox1/α-CD30 (C) or α-Prox1/α-CD68 (D). The blood vessels are surrounded by lymphatic vessels as shown by green staining (Prox1). (E) tissue section of CAM-lymphoma tissue stained with α-Prox1/α-CD30 revealing lymphoma cells inside lymphatic vessels (white arrows). Scale bar: 140 µm.


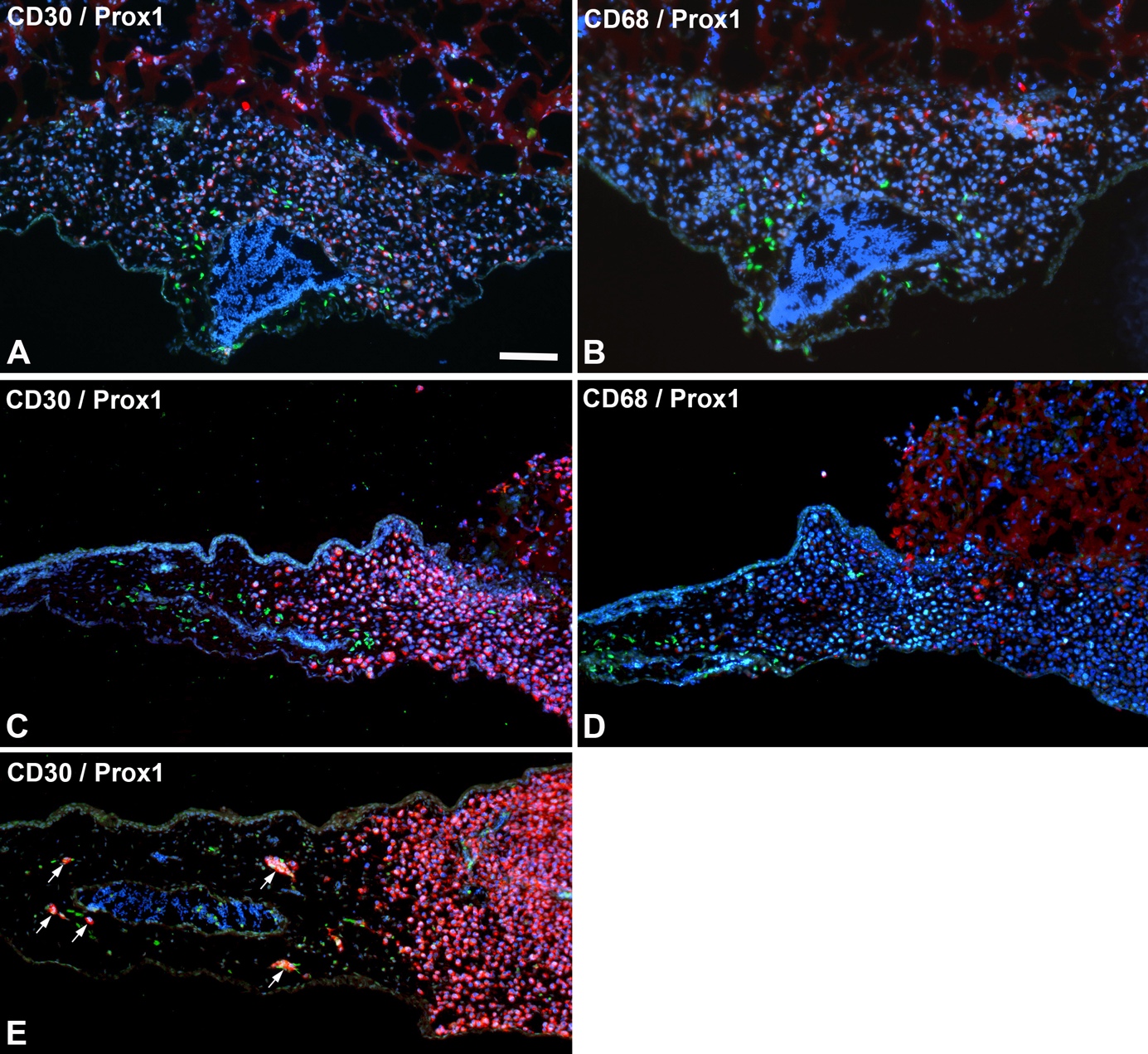


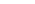


**Supplemental Figure 8:** **CD163 and CD206 double staining in a representative tonsil and HL tissue sections.**

Immunostaining of CD163 labeled with Alexa 488 and CD206 labeled with Alexa 555 (**A**) Tonsil tissue showing CD163 (green) and CD206 (red) positive cells mainly around germinal centers. Notice: red fluorescence between adjacent germinal centers represents auto-fluorescent erythrocytes and positively stained endothelium. (**B**) Detail of a germinal center from **A** (white rectangle in **A**). Only very few scattered Mφ are present within the germinal center. (**C-D**) Detailed comparison of the two markers (white rectangle in **B**). (**C)** CD163-positive Mφ (green channel, green arrowheads) and (**D**) CD206 positive Mφ (red channel, red arrowheads). Double stained Mφ are indicated by yellow arrows. **(E**) Tissue from a HL biopsy showing the variable pattern of CD163^+^ and CD206^+^ cells. (**F**) Detail of **E** (white rectangle in **E**). (**G-H**) Comparison of the two markers in detail (white rectangle in F). (**G**) CD163^+^ Mφ (green channel) and (**H**) CD206^+^ Mφ (red channel). Double stained macrophages are shown in both channels. DAPI staining of nuclei (blue) serves as navigation reference. Scale bars: (**A**, **E**) 500 µm; (**B**, **F**) 100 µm; (**C**, **D**, **G**, **H**) 50 µm.


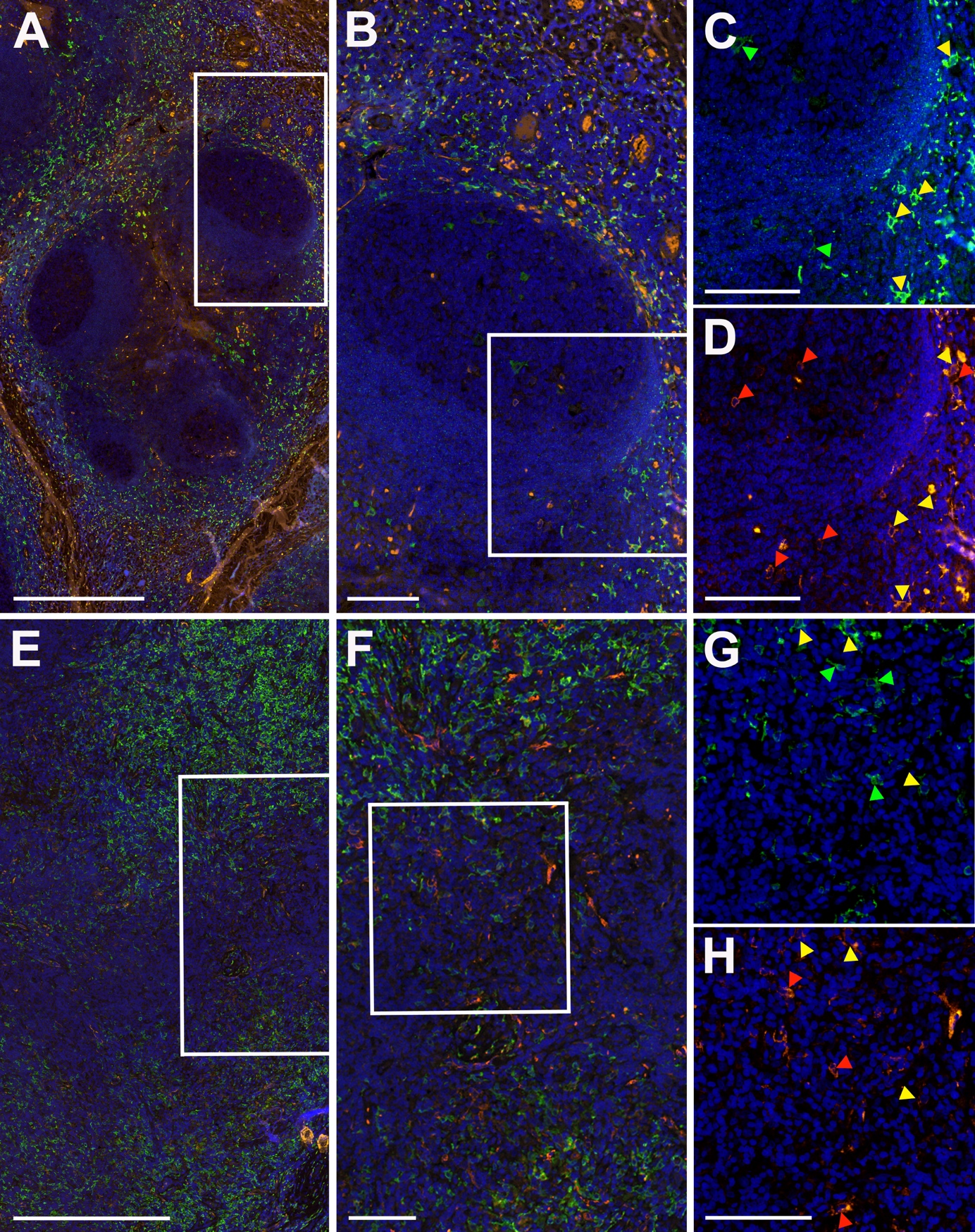


**References**

1. Luo W, Friedman MS, Shedden K, Hankenson KD, Woolf PJ. GAGE: generally applicable gene set enrichment for pathway analysis. 2009 [cited 2019 Oct 30]; Available from: http://www.biomedcentral.com/1471-2105/10/161

2. Luo W, Brouwer C. Pathview: an R/Bioconductor package for pathway-based data integration and visualization. Bioinformatics [Internet]. Narnia; 2013 [cited 2019 Oct 30];29:1830–1. Available from: https://academic.oup.com/bioinformatics/article-lookup/doi/10.1093/bioinformatics/btt285

3. Saccomano M, Albers J, Tromba G, Dobrivojević Radmilović M, Gajović S, Alves F, et al. Synchrotron inline phase contrast µCT enables detailed virtual histology of embedded soft-tissue samples with and without staining. J Synchrotron Radiat [Internet]. International Union of Crystallography; 2018 [cited 2019 Apr 15];25:1153–61. Available from: http://scripts.iucr.org/cgi-bin/paper?S1600577518005489

4. Cunningham WP, Crane FL. Variation in membrane structure as revealed by negative staining technique. Exp Cell Res [Internet]. Academic Press; 1966 [cited 2019 Apr 15];44:31–45. Available from: https://www.sciencedirect.com/science/article/pii/0014482766904101
